# Host factors that promote retrotransposon integration are similar in distantly related eukaryotes

**DOI:** 10.1371/journal.pgen.1006775

**Published:** 2017-12-12

**Authors:** Sudhir Kumar Rai, Maya Sangesland, Michael Lee, Caroline Esnault, Yujin Cui, Atreyi Ghatak Chatterjee, Henry L. Levin

**Affiliations:** Section on Eukaryotic Transposable Elements, Division of Molecular and Cellular Biology, Eunice Kennedy Shriver National Institute of Child Health and Human Development, National Institutes of Health (NIH), Bethesda, Maryland, United States of America; Fred Hutchinson Cancer Research Center, UNITED STATES

## Abstract

Retroviruses and Long Terminal Repeat (LTR)-retrotransposons have distinct patterns of integration sites. The oncogenic potential of retrovirus-based vectors used in gene therapy is dependent on the selection of integration sites associated with promoters. The LTR-retrotransposon Tf1 of *Schizosaccharomyces pombe* is studied as a model for oncogenic retroviruses because it integrates into the promoters of stress response genes. Although integrases (INs) encoded by retroviruses and LTR-retrotransposons are responsible for catalyzing the insertion of cDNA into the host genome, it is thought that distinct host factors are required for the efficiency and specificity of integration. We tested this hypothesis with a genome-wide screen of host factors that promote Tf1 integration. By combining an assay for transposition with a genetic assay that measures cDNA recombination we could identify factors that contribute differentially to integration. We utilized this assay to test a collection of 3,004 *S*. *pombe* strains with single gene deletions. Using these screens and immunoblot measures of Tf1 proteins, we identified a total of 61 genes that promote integration. The candidate integration factors participate in a range of processes including nuclear transport, transcription, mRNA processing, vesicle transport, chromatin structure and DNA repair. Two candidates, Rhp18 and the NineTeen complex were tested in two-hybrid assays and were found to interact with Tf1 IN. Surprisingly, a number of pathways we identified were found previously to promote integration of the LTR-retrotransposons Ty1 and Ty3 in *Saccharomyces cerevisiae*, indicating the contribution of host factors to integration are common in distantly related organisms. The DNA repair factors are of particular interest because they may identify the pathways that repair the single stranded gaps flanking the sites of strand transfer following integration of LTR retroelements.

## Author summary

Retroviruses and retrotransposons are genetic elements that propagate by integrating into chromosomes of eukaryotic cells. Genetic disorders are being treated with retrovirus-based vectors that integrate corrective genes into the chromosomes of patients. Unfortunately, the vectors can alter expression of adjacent genes and depending on the position of integration, cancer genes can be induced. It is therefore essential that we understand how integration sites are selected. Interestingly, different retroviruses and retrotransposons have different profiles of integration sites. While specific proteins have been identified that select target sites, it’s not known what other cellular factors promote integration. In this paper, we report a comprehensive screen of host factors that promote LTR-retrotransposon integration in the widely-studied yeast, *Schizosaccharomyces pombe*. Unexpectedly, we found a wide range of pathways and host factors participate in integration. And importantly, we found the cellular processes that promote integration relative to recombination in *S*. *pomb*e are the same that drive integration of LTR-retrotransposons in the distantly related yeast *Saccharomyces cerevisiae*. This suggests a specific set of cellular pathways are responsible for integration in a wide range of eukaryotic hosts.

## Introduction

Retroviruses integrate their DNA sequence into the chromosomes of infected cells to achieve permanent and reliable replication. A substantial amount of biochemical and genetic information is known about the catalysis of integration and the host factors responsible for the virus specific positions of integration [1–3]. The bulk of information about the factors required for integration is derived from high throughput sequencing of insertion profiles. Specific patterns of integration such as the promoter sequences selected by gamma retroviruses or the actively transcribed genes selected by lenti-retroviruses, result from direct interactions between the viral integrase and chromosome bound host proteins [3]. These diverse patterns of integration suggest the host pathways that promote integration are virus specific. This understanding remains to be tested since genome-wide siRNA screens for host factors have only been performed for HIV-1 infection and the complexity of these results provided little consensus [4–7].

Long terminal repeat (LTR) retrotransposons are mobile elements that are the progenitors of retroviruses [8, 9] and are studied extensively as important models for retrovirus replication [10–13]. LTR-retrotransposons model the same processes of particle formation, reverse transcription and integration that are central to retrovirus propagation. One advantage of LTR-retrotransposons is they are highly active in well-characterized model organisms, *Saccharomyces cerevisiae* and *Schizosaccharomyces pombe*. Extensive study of these model systems has resulted in significant understanding of particle formation, reverse transcription, and integration [10–13].

Genetic assays that measure retrotransposon mobility rely on single copy elements tagged with a drug resistance gene or on plasmids that express retrotransposon mRNA [14–17]. These assays were used with collections of deletion strains or insertion mutants to identify host factors important for transposition of Ty1, and Ty3 in *S*. *cerevisiae* [18–22] and extensive screens were performed to identify host factors that restrict transposition in *S*. *cerevisiae* [20, 23, 24]. Host factors important for transposition are involved in chromatin modification, transcription, translation, vesicle trafficking, nuclear transport, and DNA repair. These genetic screens provide a broad view of what cellular systems support transposition in *S*. *cerevisiae*. However, it is not known how general these processes are in supporting transposition in other eukaryotes. More importantly, none of these screens were designed to identify host factors that promote integration.

*S*. *pombe* is distantly related to *S*. *cerevisiae* having diverged approximately 350 million years ago [25–27]. The identification of host factors in *S*. *pombe* important for retrotransposition would provide a valuable means for determining whether the cellular processes that support retrotransposition are conserved between distantly related eukaryotes.

A significant body of research on the LTR-retrotransposon Tf1 of *S*. *pombe* describing protein expression, particle assembly, reverse transcription, and transposition activity has established Tf1 as a valuable model system [12]. The transposition of Tf1 in *S*. *pombe* is measured by expressing a drug resistant copy of Tf1 from a multi-copy plasmid [14, 28, 29]. This genetic assay combined with high throughput sequencing shows that Tf1 has a pronounced pattern of integration that favors the promoters of stress response genes [30, 31]. Recent studies revealed that the DNA binding protein Sap1 plays an important role in directing integration to stress response promoters [32, 33]. Although two-hybrid assays detected interaction between Sap1 and IN, biochemical and immunoprecipitation experiments fail to detect this interaction [32, 33]. We therefore believe other factors necessary for integration bridge the Sap1-IN interaction. To identify potential bridging proteins we applied a genome-wide screen for factors involved in integration. For this, we applied a unique combination of assays that together detect defects in integration. We identified a set of 61 host factors that promote integration relative to recombination and participate in key cellular processes such as transcription, chromatin structure, mRNA processing, translation, vesicle trafficking, and DNA repair. With these results we discovered there is a surprising diversity in processes involved in integration. Although it’s not clear with this type of genetic screen which factors impact integration directly, we found strong similarity in the host factors that promote integration in distantly related eukaryotes.

## Results and discussion

To identify host factors important for the integration, we measured transposition frequencies in 3,004 deletion strains of *S*. *pombe* that have single non-essential genes replaced with *neo* [34]. We monitored transposition in these strains with a plasmid that expressed Tf1. Previous studies of Tf1 activity relied on expression of Tf1 with a copy of *neo* inserted in a non-coding site of the element [14, 35]. Because the deletion strains all contain *neo*, we replaced the *neo* selection marker in Tf1 with *nat*, a gene that provides resistance to nourseothricin (Nat) (S1 Fig).

The goal of our screen was to identify genes that specifically contribute to the mechanism of integration. For this purpose we screened each deletion strain with a transposition assay and a related assay that measures homologous recombination between Tf1 cDNA and the Tf1 expression plasmid [36]. Homologous recombination will be low in deletion strains with reduced cDNA caused by defects in early stages of transposition such as expression of Tf1 protein, assembly of virus-like particles, or transport of the integrase and cDNA into the nucleus. By identifying deletions that reduced transposition but did not lower recombination of cDNA with plasmid sequence, we could generate a list of candidate factors that specifically promoted integration [36].

**Fig 1:**
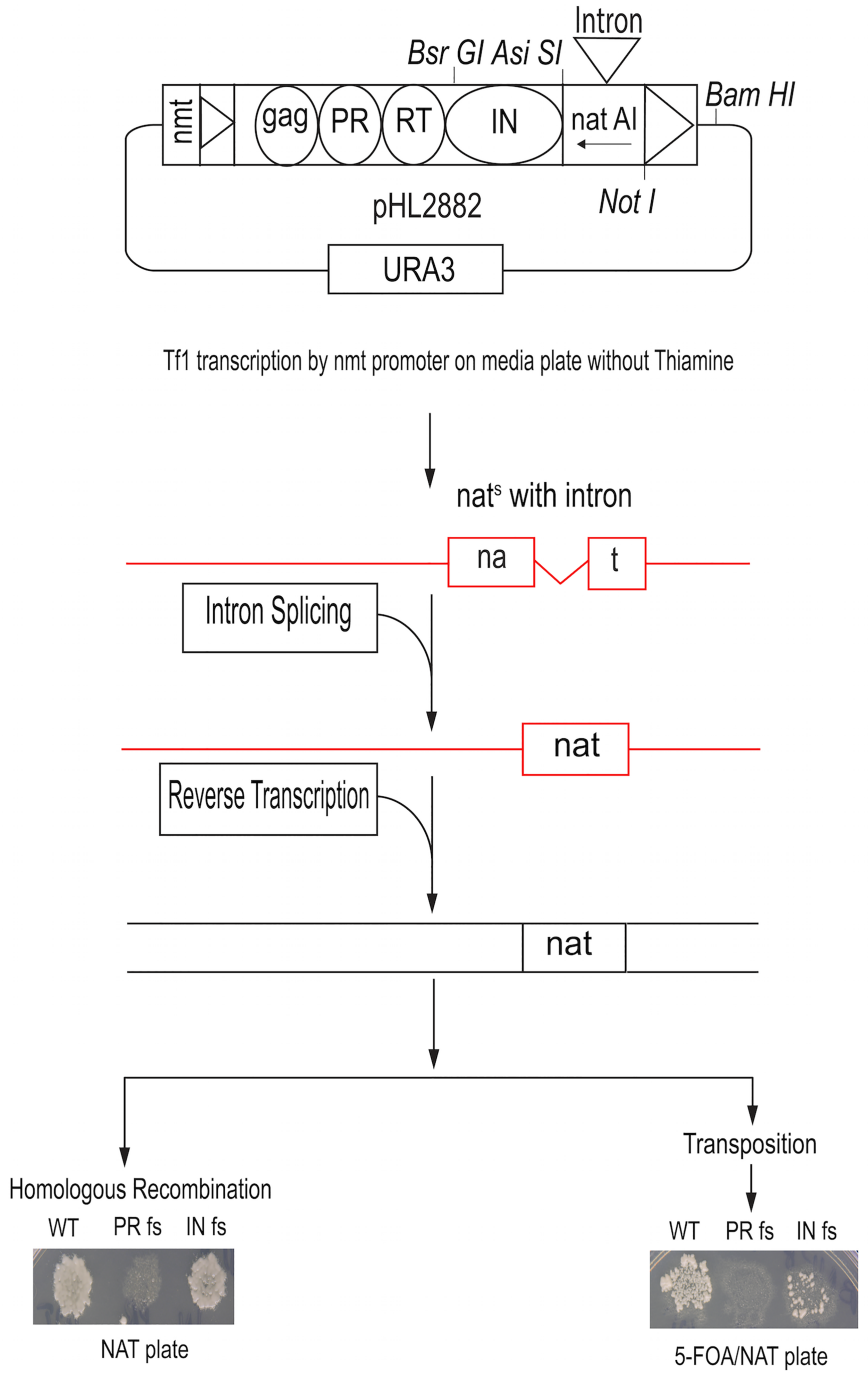
Assays that measure Tf1 transposition and homologous recombination of cDNA detect defects in integration. Transposition is detected by expressing Tf1-*nat*AI in cells on agar plates and replica printing patches of cells to medium containing FOA and Nat. The intron in *nat* is spliced out, the mRNA (red) is reverse transcribed, and IN inserts Tf1 cDNA with an active *nat* into *S*. *pombe* chromosomes. Frame shift mutations at the N termini of PR (PRfs) and IN (INfs) greatly reduce transposition (right panel). Tf1 cDNA is detected in the nucleus by replica printing cell patches to medium containing Nat (left panel).

The measure of homologous recombination of Tf1 cDNA with the expression plasmid relies on *nat* disrupted by an artificial intron (AI). Recombination results in resistance to Nat because the intron is spliced from the Tf1 mRNA (Materials and methods) (Fig 1). For measures of transposition, the expression plasmid is removed, and cells with integration become Nat resistant (Fig 1). A frameshift mutation in IN (INfs) disrupts integration but allows low levels of recombination between cDNA and genomic copies of the closely related Tf2 (Fig 1) [36]. A frameshift in Tf1 protease (PRfs) that blocks expression of RT and IN results in no resistance to Nat. It is notable that in the cDNA recombination assay INfs does not significantly reduce resistance to Nat as expected since the homologous recombination is independent of integration (Fig 1). We note that Tf1 is unique in that reverse transcription is independent of IN. Clear evidence shows INs of Ty1, Ty3 and retroviruses are required for production of cDNA [37, 38] [39] [40]. This feature of Tf1 provides an opportunity to study effects on integration independent of the requirements for reverse transcription.

### Genetic screen of deletion strains for defects in integration

**Fig 2:**
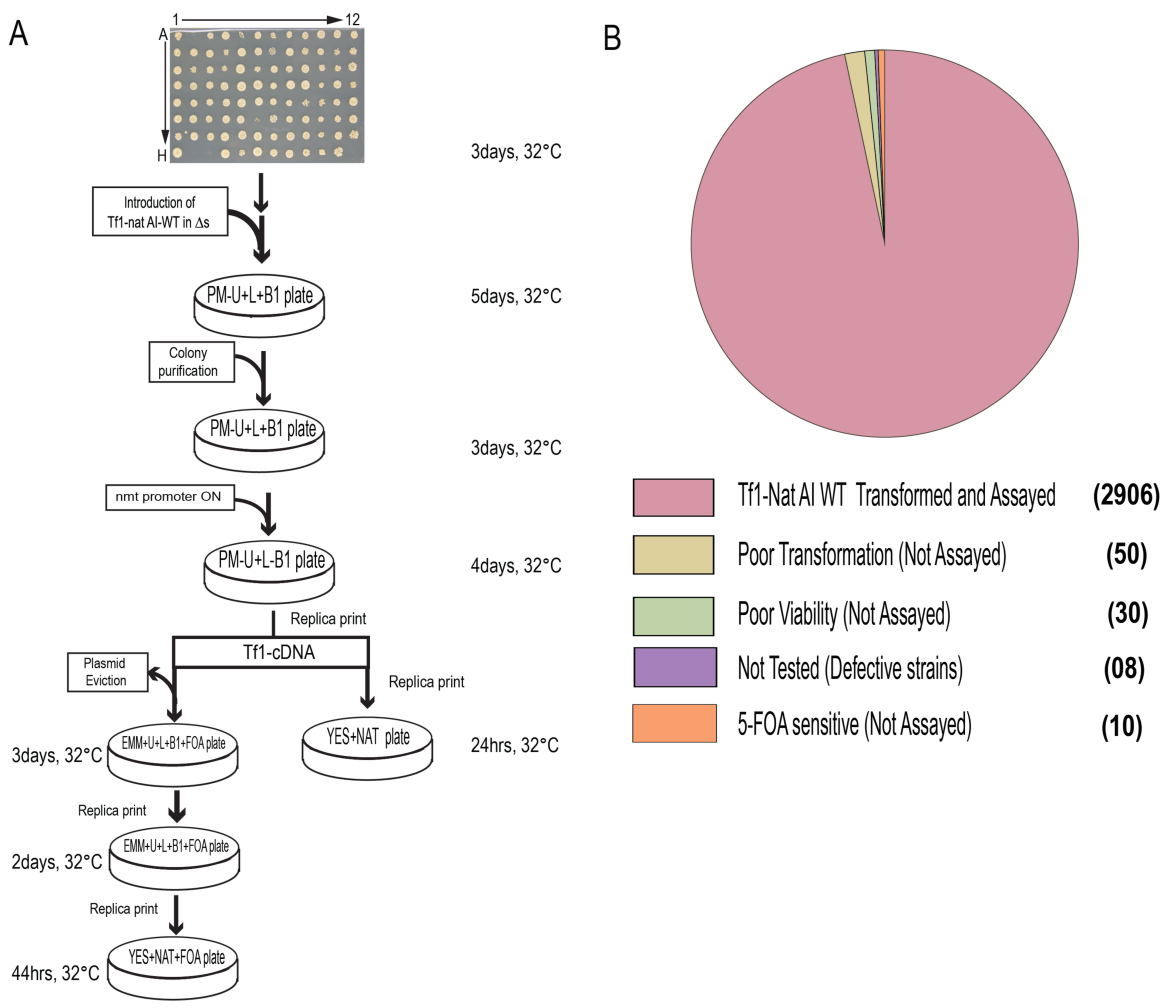
A high throughput screen measured transposition and homologous recombination of deletion strains. A. Strains in the deletion library (ΔS) in 96-well format were grown on agar and the Tf1-*nat*AI expression plasmid was introduced into four isolates of each deletion strain by selecting for growth on minimal medium plus amino acids and vitamin B1 lacking uracil (PM-U+L+B1). Patches are replica printed to medium lacking vitamin B1 (PM-U+L-B1) to induce expression of Tf1-*nat*AI. The induction plates are subsequently replica printed to YES+Nat medium to detect recombination and to minimal medium with FOA and Nat (EMM+U+L+B1+FOA+Nat) to detect transposition. B. Deletion strains that could not be assayed for transposition or recombination had poor transformation frequency, poor viability, contained genetic defects, or were sensitive to FOA. The numbers of strains with these properties are shown in parenthesis.

The haploid deletion strains of the Bioneer 2.0 collection were individually transformed to introduce the Tf1-*nat*AI plasmid (Fig 2A). Fifty strains did not grow on plates lacking uracil, which was used to select for uptake of the plasmid (Fig 2B, S1 Table). While some of these strains contained deletions in uracil catabolism genes other deletion strains had very slow growth rates, might be unable to tolerate the lithium treatment of transformation, or might be incapable of transferring the plasmid DNA into the nucleus. Despite the strains that were poor growers or transformation defective, the expression plasmid was successfully introduced into 2,954 deletion strains. For each of these strains, four independent isolates containing the plasmid were assayed for transposition and recombination activities as diagramed in Fig 2A and listed in S1 Table.

**Fig 3:**
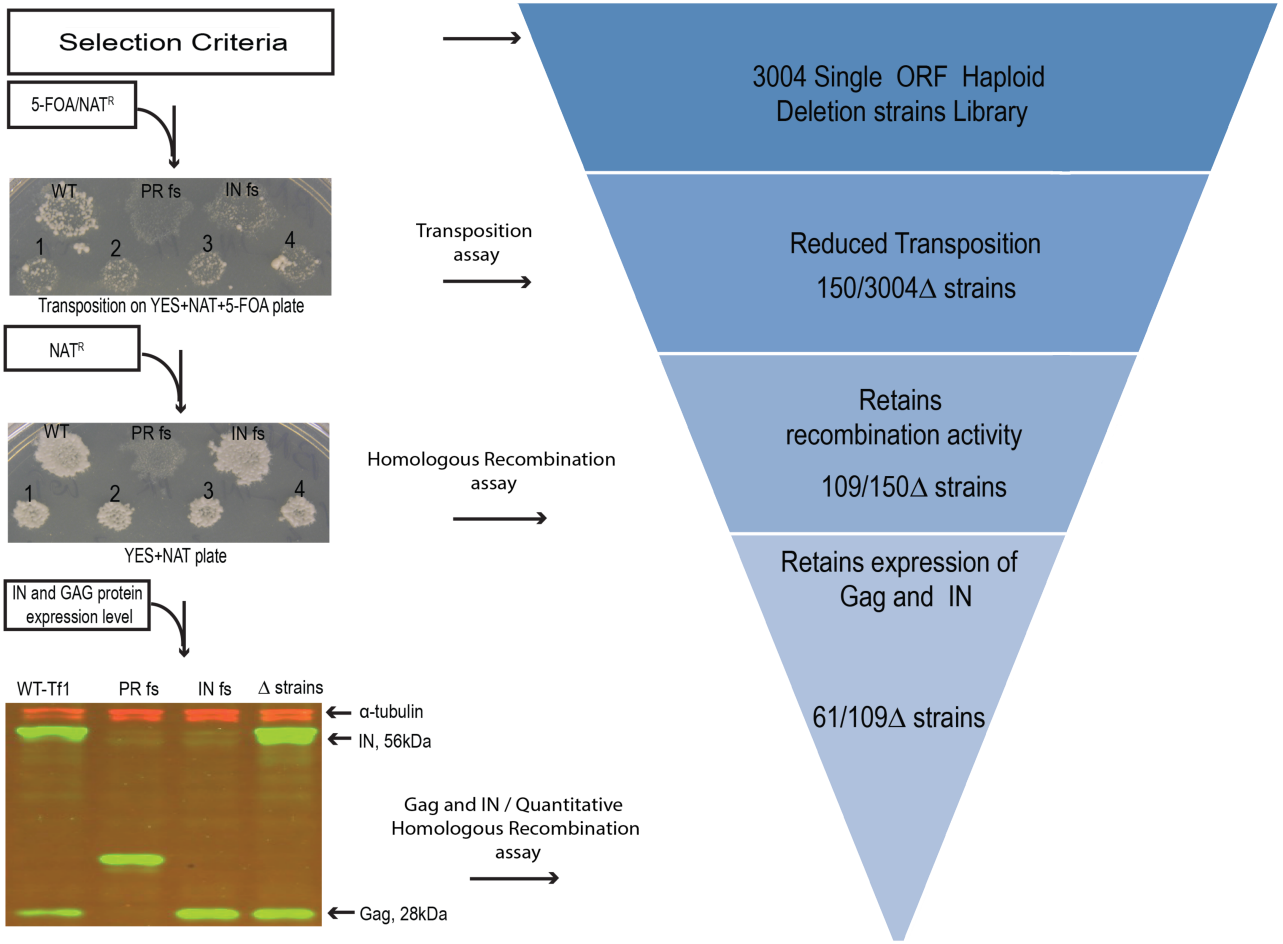
Genes that promote Tf1 integration were identified by screening deletion strains with assays that measure transposition, homologous recombination, and expression of Tf1 protein. Out of 150 strains with low transposition, 109 supported high levels of homologous recombination. These candidates were further analyzed with a quantitative recombination assay to detect reduced cDNA in the nucleus and with immunoblots to detect reduced levels of Gag and IN.

All four independent isolates of each deletion strain were scored for transposition activity on a scale of 0 to 5 by comparing growth to wild-type strains of *S*. *pombe* which received a score of 5 (S2 Fig and S1 Table). Ten deletion strains were unable to grow on FOA-containing medium and therefore could not be scored (Fig 2B and S1 Table). Additional strains that could not be scored include 30 deletions that had poor viability, and eight strains with unidentified genetic defects (S1 Table). A total of 150 deletion strains had a significant defect in transposition frequency and were scored 2.5 or lower (Fig 3 and S1 Table).

For the recombination assays the patches were also scored on a scale of 0 to 5 where wild-type was assigned the score of 5 (S3 Fig). A total of 183 deletion strains exhibited a notable defect in homologous recombination and were scored lower than 4 (S1 Table). Of the 150 deletion strains with low levels of transposition 41 also exhibited recombination activity lower than 4 indicating these genes were important for intermediate stages of transposition such as particle assembly, reverse transcription or nuclear import (S2 Table). For example, deletion of *nup124* resulted in low recombination and transposition, a result previously described in studies that found Nup124 interacts with Gag and promotes nuclear import of Tf1 protein and cDNA [41–46]. Importantly, we identified 109 deletion strains that had strong homologous recombination scores (4 or higher) but had significantly reduced transposition activities scoring 2.5 or less (Fig 3, S1 Table). These strains represented our initial list of candidates that could be important for integration (S3 Table).

### Quantitative recombination assay as a sensitive measure for reduced levels of cDNA in the nucleus

One concern with our list of integration deficient candidates was that the homologous recombination assay relied on the growth of cells in patches and reductions of two to four-fold in the growth of a patch is not reliably detected. To test whether integration deficient candidates had reductions in recombination not observed with patches we screened the integration candidates with a quantitative assay that measures the fraction of cells in liquid cultures that have recombination events (S4 Fig) [29]. Each deletion strain was assayed in triplicate and each replica was an independent plasmid containing isolate (S3 Table). The results of this assay were highly reproducible.

**Fig 4:**
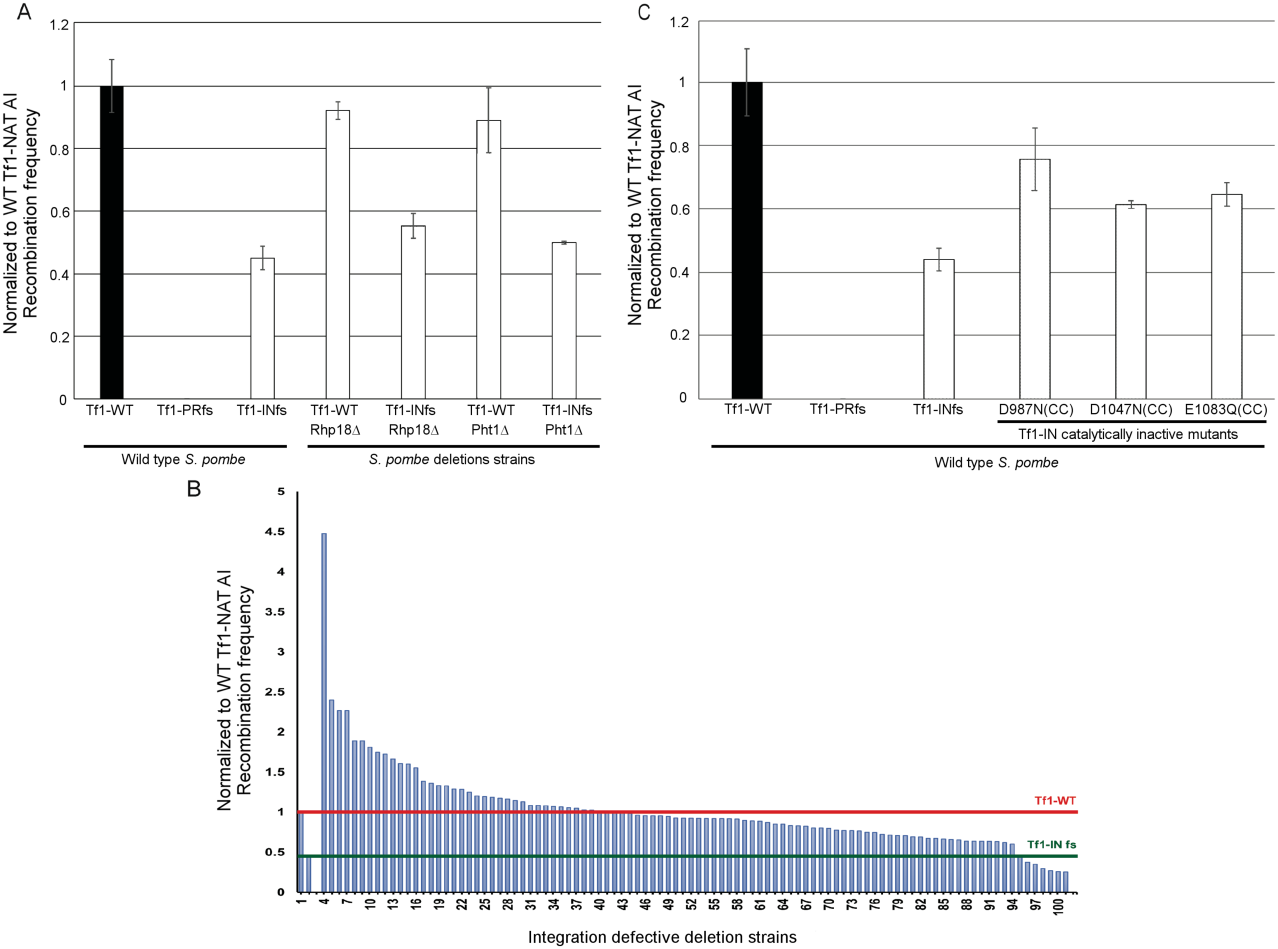
The quantitative homologous recombination assay detected deletion strains with reduced recombination that was not detected with the patch assay. A. Quantitative recombination assays of deletion strains expressing wild-type Tf1-*nat*AI and the INfs. B. Quantitative recombination frequencies are shown in a histogram of strains sorted from highest to lowest. The numbers on the x-axis identify strains in S3 Table. The deletion strains here were shown by the yeast patch assays to have defects in transposition but not homologous recombination (S3 Table). The red line illustrates the homologous recombination activity of wild-type Tf1 in wild-type *S*. *pombe*. The green line shows the homologous recombination activity of the INfs in Wild-type *S*. *pombe*. C. Quantitative homologous recombination assays of cells with catalytically inactive mutants in the catalytic core (CC) of IN.

Although the homologous recombination activity of Tf1 looks to be independent of integration as Tf1 lacking IN (Tf1 INfs) has approximately the same amount of activity as Tf1 with IN (Fig 1), quantitative measures show that approximately 50% of the recombination response is IN dependent [29, 36]. The quantitative recombination assays reported here confirmed this finding that with Tf1-*nat*AI the INfs reduced recombination activity to 45% (SD 4.0%) of wild-type Tf1-*nat*AI (Fig 4A and S3 Table). Therefore, deletion strains with reduced integration but intact homologous recombination would be expected to exhibit the same recombination levels as the INfs, 45%.

Surprisingly, 91 of the integration deficient candidates possessed recombination activity greater than exhibited by the INfs (Fig 4B and S3 Table). This high number of deletions that had more recombination than the INfs suggests that in the absence of integration the IN protein might promote homologous recombination of the cDNA. This was tested by measuring recombination frequencies of deletions in *rhp18* and *pht1* with the INfs. In addition to showing high levels of cDNA recombination, these two genes were selected because of interesting potential roles in Tf1 integration as described below. While Tf1-*nat*AI expressed in these strains produced recombination activities higher than the INfs in wild-type cells, expression of INfs in the deletion strains resulted in reduction in recombination activity to levels similar in wild-type *S*. *pombe* containing INfs (Fig 4A). These results indicate that the presence of IN stimulates cDNA recombination independent of IN catalysis. Wild-type strains with single amino acid substitutions in catalytic residues of IN had recombination activities averaging 67% of the strain with intact IN (Fig 4C). Since the catalytic mutations disrupted integration without reducing levels of IN protein (S5 and S6 Figs), the 67% recombination vs. the 45% of the INfs indicates that IN lacking catalytic activity does stimulate homologous recombination. It is possible this occurs because IN protects cDNA from degradation. In considering these IN contributions to recombination we chose the average activity of the catalytically inactive IN mutants, 67% (Fig 4C) as the level for recombination activity expected in the absence of integration. Deletion strains with recombination levels below 60% were deemed to have a defect in Tf1 recombination. By this criterion 8 of the integration deficient candidates had reduced homologous recombination (S3 Table) and were excluded from the final list of candidates (Table 1).

**Table 1:** Host factors that function in Tf1 integration.

**Non-chromatin associated factors**			
Biological function	Systematic gene ID	Protein	Gene Product
Nuclear transport			
	SPCC18B5.07c	Nup61	nucleoporin Nup61
	SPCC1753.05	Rsm1	RNA export factor Rsm1
	SPBC1703.03c	Syo2	armadillo repeat protein, involved in nucleocytoplasmic transport Syo2 (predicted)
Protein synthesis, mRNA processing			
	SPAC30C2.04	Asc1	cofactor for cytoplasmic methionyl-and glutamyl-tRNA synthetases Asc1 (predicted)
	SPBC947.10	Dsc1	proposed involvement in the quality control of misfolded transmembrane containing proteins
	SPAC343.10	met11	methylenetetrahydrofolate reductase Met11
	SPAC1610.02c	Mrpl1	mitochondrial ribosomal protein subunit L1 (predicted)
	SPBC19G7.10c	Pdc2	topoisomerase II-associated deadenylation-dependent mRNA-decapping factor Pdc2 (predicted)
	SPCC24B10.09	Rps1702	40S ribosomal protein S17 (predicted)
	SPBC1709.09	rrf1	mitochondrial translation termination factor Rrf1
	SPCC1919.05	ski3	Ski complex TPR repeat subunit Ski3 (predicted)
	SPCC162.12	Tco89	TORC1 subunit Tco89
Vesicle transport (ER to Golgi, ESCRT)			
	SPBC725.10		mitochondrial transport protein, tspO homolog (predicted
	SPAC16A10.03c		Ubiquitin-protein ligase E3Pep5/vps11 like
	SPBC1539.08	Arf6	ADP-ribosylation factor, Arf family
	SPAC18G6.10	Lem2	LEM domain protein
	SPAC30.01c	Sec72	Sec7 domain protein, ARF GEF
	SPAC31A2.13c	Sft1	SNARE Sft1 (predicted)
	SPBC215.14c	Vps20	ESCRT III complex subunit
	SPAC1142.07c	Vps32	ESCRT III complex subunit
Ubiquitin-mediated proteolysis			
	SPAPB17E12.04c	Csn2	COP9/signalosome complex subunit
	SPAC6C3.08	Nas6	proteasome regulatory particle, gankyrin (predicted)
	SPCC338.16	Pof3	F-box protein, ubiquitin ligase
	SPCC188.08c	Ubp5	ubiquitin C-terminal hydrolase
Signal transduction			
	SPCC285.09c	Cgs2	cAMP-specific phosphodiesterase
	SPBP23A10.10	Ppk32	serine/threonine protein kinase (predicted)
	SPBC646.13	Sds23	PP2A-type phosphatase inhibitor
Metabolism			
	SPBC1861.05		pseudouridine-metabolizing bifunctional protein (predicted)
	SPCC594.04c		steroid oxidoreductase superfamily protein (predicted)
	SPBC21C3.08c	Car2	ornithine transaminase Car2, L-proline biosynthetic process
	SPAC1805.06c	Hem2	porphobilinogen synthase (predicted)
	SPCC794.12c	Mae2	Malate dehydroxgenase, oxaloacetate decarboxylating
	SPBC26H8.01	Thi2	thiazole biosynthetic enzyme
	SPAC19G12.15c	tpp1	trehalose-6-phosphate phosphatase
	SPAC3G6.09c	tps2	trehalose-phosphate synthase (predicted)
Kinetochore			
	SPBC2G2.14	Csi1	mitotic chromosome segregation protein
	SPCCC576.12c	Mhf2	Kinetochore Protein, CENP-X Ortholog, FANCM-MHF complex subunit
	SPCC1442.02		central kinetochore associated family protein (predicted)
Cytoskeleton			
	SPBC359.06	Mug14	ubiquitously expressed cytoskeletal adducin
Unknown function			
	SPAC7D4.03c		conserved fungal family
**Chromatin associated factors**			
Chromatin			
	SPBC36B7.08c		nucleosome assembly protein (predicted)
	SPCC24B10.19c	Nts1	Clr6 histone deacetylase complex subunit
	SPBC11B10.10c	Pht1	histone H2A variant H2A.Z
	SPCC306.04c	Set1	histone lysine methyltransferase
	SPAC2F7.08c	Snf5	SWI/SNF complex subunit
	SPAC13A11.04c	Ubp8	SAGA complex ubiquitin C-terminal hydrolase
Transcription			
	SPCC757.04		transcription factor (predicted)
	SPAC1851.03	Ckb1	CK2 family regulatory subunit
	SPAC1D4.11c	Lkh1	dual specificity protein kinase
	SPAC31G5.12c	Maf1	repressor of RNA polymerase III
	SPAC664.03	Paf1	RNA Pol II associated Paf1 complex
	SPBC12D12.06	Srb11	cyclin C, Srb mediator subunit
	SPAC20H4.03c	Tfs1	transcription elongation factor TFIIS
Splicing			
	SPBC32F12.05c	Cwf12	subunit of the NineTeen splicing complex
	SPBC2A9.11c	Iss9	Possibly involved in splicing (predicted)
	SPCC825.05c	Pwi1	splicing coactivator SRRM1 (predicted)
	SPBC19C2.14	Smd3	Core Sm protein associated with snRNPs
DNA repair			
	SPAC1556.01c	Rad50	DNA repair protein
	SPAC644.14c	Rad51	RecA family recombinase
	SPBC1734.06	Rhp18	Rad18 homolog ubiquitin protein ligase E3,
	SPBC2D10.12	Rhp23	Rad23 homolog

### Expression levels of Gag and IN

Another question when validating candidate strains was whether the deletion mutations reduced the levels of Tf1 proteins. We addressed this possibility for the primary set of 101 integration deficient candidates by performing quantitative immunoblotting of whole cell extracts (Materials and methods) (Fig 3 and S3 Table) [47]. Candidate strains with reduced Gag or IN levels by two-fold or greater were considered to have poor expression of Tf1 protein and as a result these factors were removed from the list of candidates that mediate integration. Results of these quantitative immunoblots identified 64 integration candidates that expressed normal IN and Gag levels (S3 Table). The majority of the candidates identified as having reduced homologous recombination also had low Tf1 protein expression. However, three deletion strains had normal levels of Gag and IN but had reduced homologous recombination, as measured with the quantitative assay. As a result our final list of candidates that impact integration had 61 factors (Table 1).

To understand how the candidate factors in our final list may contribute to integration, we grouped them by biological function encompassing two broad categories; non-chromatin associated and chromatin associated processes (Table 1) [48, 49].

### Non-chromatin associated host factors that promote integration

Factors that lack association with chromatin are less likely to participate directly in integration. Nevertheless, 40 of the 61 candidates with defects in integration have no established association with chromatin (Table 1). These non-chromatin associated proteins function in nuclear transport, protein synthesis, mRNA processing, vesicle transport, ubiquitination, signal transduction, metabolic processes, chromosome segregation, and cytoskeleton structure.

Among the list of non-chromatin associated factors that promote Tf1 integration are three nuclear transport proteins, Nup61, Rsm1, and Syo2. Previous studies found several nuclear pore factors contribute to LTR-retrotransposon activity by mediating transport of transposon factors and cDNA into the nucleus (Table 2) [18–20, 22, 46]. Nuclear pore factors also mediate the replication of retroviruses by mediating nuclear entry of the IN complexes [50–54]. However, these functions would not be expected to mediate integration as nuclear pores are imbedded in the nuclear envelope. The contribution of Nup61 to Tf1 integration could be indirect by transporting other factors that mediate integration. However, there is evidence that some nuclear pore complexes can interact directly with chromatin [55–57] and in the case of HIV-1, integration appears to favor the nuclear periphery [53]. It is notable that Nup61 is a homolog of Nup2, which in *S*. *cerevisiae* binds to a set of promoters and activates gene expression [58–60]. Other studies found that tRNA genes localize at nuclear pore complexes of *S*. *cerevisiae* via an interaction between DNA sequence and Nup2 [61]. These results suggest the possibility that Nup61 could bind promoters in *S*. *pombe* and directly stimulate Tf1 integration. This possibility also exists for nuclear pore factors that promote Ty1 and Ty3 transposition in *S*. *cerevisiae*. Deletion of five different nuclear pore factors inhibits Ty1 transposition and deletion of *NUP59* results in reduced Ty3 transposition. In all these cases cDNA production is not reduced suggesting the Nups may contribute to integration in *S*. *cerevisiae* (Table 2).

**Table 2:** Factors that promote transposition.

Function	Tf1 integration in *S*. *pombe*	Ty1 transposition in *S*. *cerevisiae*	Ty3 transposition in *S*. *cerevisiae*	Features that promote transposition in *S*. *cerevisiae* and *S*. *pombe*
Nuclear transport	Nup61	Nup84^1^^,^^2^^#^, Nup120^3^^#^, Nup133^1^*^,^^3^^#^, Nup170^2^^,^^3^^#^, Nup188^3^^#^	Nup59^4^^#^, Nup157^5^*	Components of the nuclear pore
Protein synthesis; mRNA processing	Asc1, Dsc1, Met11, Mrpl1, Rps1702, Rrf1, Tco89; Pdc2, Ski3,	Bud21^3^*, Dbp3^1^^#^, Dbp7^3^*, eIF2A^3^^#^, Hcr1^3^*, Loc1^3^*, Lst7^3^*, Mrpl39^3^^#^, Mrpl49^3^^#^, Mrpl7^3^^#^, Mrpl8^3^^#^, Mrps28^3^^#^, Mrt4^3^*, Rkm4^3^*, Rpl14a^1^^#^, Rpl15b^2^, Rpl16b^1^^#^^,^^3^^#^, Rpl18a^2^, Rpl19a^3^*, Rpl19b^1^*, Rpl1b^2^, Rpl20b^1^*, Rpl21a^2^, Rpl21b^1^^#^, Rpl27a^3^*^,^^2^, Rpl2b^2^, Rpl31a^3^*, Rpl33b^3^^#^, Rpl34a^3^^#^, Rpl37a^3^^#^, Rpl39^2^, Rpl40a^2^, Rpl41b^2^, Rpl43a^3^*, Rpl6a^1^*, Rpl7a^3^*, Rpp1a^1^^#^^,^^3^^#^, Rpp2b^2^, Rps0b^2^, Rps10a^1^*^,^^2^, Rps11a^3^^#^, Rps19a^3^^#^, Rps19b^3^*^,^^2^, Rps25a^3^*^,^^2^, Rps27b^3^^#^, Rps30a^3^*, Rsa3^3^^#^, Rsm25^2^, Sqs1^3^^#^, Utp30^3^^#^; Caf40^3^^#^, Cbc2^1^*, Ccr4^3^^#^, Cth1^3^*, Dbr1^1^*, Dhh1^3^*, Lea1^1^^#^, Lsm1^1^*^,^^3^, Lsm6^3^^#^, Mot2^2^, Mpp6^3^^#^, Mrt4^3^*, Nop12^1^^#^, Pol32^3^^#^, Pop2^1^*, Ref2^3^*, Rit1^1^^#^^,^^2^, Rpb4^3^^#^, Rrp6^2^, Rrp8^2^, Ski8^3^^#^, Slm3^1^^#^, Sto1^1^^#^, Trf5^3^^#^, Upf1^3^*, Upf3^3^*,	Acs1^4^^#^, Gcn20^4^^#^, Gtr1^5^^#^ Rpl6a^4^*; Dbp3^4^^#^, Dbr1^4^, Deg1^4^^#^, Dhh1^4^^#^, Kem1^4^^#^,	Ribosome subunits and RNA processing factors.
Vesicle transport	Arf6, Lem2, Sec72, Sft1, SPAC16A10.03c, SPBC725.10, Vps20, Vps32,	Apl5^1^^#^, Bro1^3^^#^^,^^5^, Erv14^1^^#^, Glo3^3^^#^, Ric1^3^^#^, Sec22^1^*, Stp22^2^, Vps9^1^*, Vps15^2^, Vps16^2^, Vps34^2^	Atg17^4^^#^, Bro1^4^^#^^,^^5^, Clc1^4^^#^, Fab1^4^^#^, Mnt4^4^*, Pep7^4^*, Rim13^4^^#^, Rim20^4^*, Snf7/Vps32^4^^#^, Snf8/Vps22^4^^#^, Vam7^4^^#^, Vph1^4^^#^, Vps20^4^^#^, Vps25^4^*, Vps27^4^*, Vps28^4^^#^, Vps36^4^^#^, Vps4^4^^#^, Vps51^4^^#^, Vps9^4^*	ESCRT complexes and vesicle transport between the ER and Golgi
Chromatin	Nts1, Pht1, Set1, Snf5, SPBC36B7.08c, Ubp8	Ard1^1^*, Dep1^1^^#^, Elf1^3^^#^, Gcn5^2^, Hda1^3^^#^, Hda3^2^^,^^3^^#^, Hda3^3^^#^, Hfi1^2^, Hmo1^3^*, Hpc2^3^^#^, Hpc2^3^^#^, Htz1^2^, Ies3^1^^#^, Isw1^3^^#^, Lge1^3^^#^, Nat1^1^*, Nat4^3^^#^, Pho23^1^^#^, Rsc2^3^^#^, Sap30^1^*, Sgf73^2^, Sin1^1^*, Sin3^1^^#^, Snf12^2^, Snf2^2^, Snf5^3^*, Snf6^3^*, Snt1^3^*, Spt10^1^*^,^^2^, Spt10^3^^#^, Spt20^2^, Spt21^1^*, Spt3^2^^,^^3^*, Spt4^1^*^,^^2^, Spt7^2^, Spt8^2^^,^^3^*, Spt8^3^*, Swi3^3^*^,^^2^, Swr1^3^^#^, Ume1^3^, Ume6^3^, Vps72^3^^#^	Eaf7^4^, Ies6^4^, Swd1^5^^#^	histone acetylation and methylation, nucleosome remodeling, and H2AZ
Transcription	Ckb1, Lkh1, Maf1, Paf1, SPCC757.04, Srb11, Tfs1,	Cst6^2^, Ctk1^1^*, Elp2^1^^#^^,^^3^, Elp3^1^^#^, Elp4^1^^#^, Elp6^1^^#^, Hac1^1^^#^, Hfi1^2^, Hmo1^2^^,^^3^*, Iki3^1^^#^, Ino2^3^, Ino4^3^^#^, Kti12^1^^#^, Med2^2^, Mig3^2^^,^^3^^#^, Pgd1^2^, Rpa49^1^*, Rpb4^3^^#^, Rpn4^2^, Rtf1^1^*, Rtg1^3^^#^, Sin4^1^*^,^^2^, Sip4^3^^#^, Spt20^2^, Spt23^3^^#^, Spt3^3^*^,^^2^, Spt7^2^, Spt8^2^, Srb8^1^^#^^,^^2^, Ssn2^1^*^,^^2^, Stb5^1^*, Sub1^1^^#^, Swi6^3^^#^, Taf14^2^, Thp2^1^*^,^^3^^#^, Tup1^2^, Usv1^3^^#^	Bas1^4^, Ctk2^4^, Sin4^4^, Ssn3^4^	mediator complex and RNA pol II elongation complexes, and transcription factors
Splicing	Cwf12, Iss9, Pwi1, Smd3	Bud31^2^, Mud2^3^^#^, Ptc1^3^^#^, Snt309^3^^#^, Snu66^2^, Sqs1^3^^#^	Sqs1^4^^#^	NineTeen Complex and snRNP factors
DNA repair	Rad50, Rad51, Rph18, Rph23	Apn1^1^*, Mms22^1^^#^^,^^3^^#^, Pms1^3^^#^, Rad16^3^^#^, Rad17^3^^#^, Rad52^1^^#^, Xrs2^1^^#^, Yen1^3^^#^	Rad24^4^^#^	Rad51-Rad52 repair complex and Mre11-Rad50-Xrs2 complex

* > 2-fold reduction in cDNA

^#^ < 2-fold reduction in cDNA

^1^ (Griffith *et al*. 2003)

^2^ (Dakshinamurthy *et al*. 2010)

^3^ (Risler *et al*. 2012)

^4^ (Irwin *et al*. 2005)

^5^ (Aye *et al*. 2004)

A collection of 10 factors that promote Tf1 integration are associated with protein synthesis and mRNA processing. While there is no unified understanding of how translation might contribute directly to integration, Risler et al. found 33 and Griffith et al. identified nine components of ribosomes or translation factors that promote Ty1 transposition [18, 22]. At least four translation factors and two translation inhibitors are involved in Ty3 transposition [11, 20, 21]. Many of the ribosome constituents and ribosome biogenesis factors that promote Ty1 and Ty3 transposition are important for translation of transposon mRNA [18, 20, 22, 62]. However, among the factors important for transposition, 20 ribosomal proteins and translation factors required for Ty1 transposition and four factors important for Ty3 transposition mediate a stage of the transposition process after reverse transcription either related to nuclear import or integration (Table 2).

Factors that supported Tf1 integration include Pdc2, and Ski3, proteins involved with deadenylation, decapping, or 3’ end mRNA degradation. Although the function of these proteins suggests they would influence Tf1 mRNA translation, the results of the homologous recombination assay indicate these mRNA stability factors impact integration. While there are no obvious means for mRNA stability to have a direct contribution to integration, factors mediating deadenylation, decapping, and 3’ mRNA decay also contribute to Ty1 (Ccr4, Lsm1, Lsm6, Ski8, Rpb4, Trf5, and Mpp6) [18] at stages after cDNA synthesis. The similarity in these factors that contribute to late stages of Tf1, Ty1 and Ty3 transposition suggests these distantly related LTR-retrotransposons may share aspects of integration that are regulated by mRNA processing and translation.

A significant cluster of host factors involved in vesicle transport was found to contribute to Tf1 integration (Table 2). This group included factors responsible for ER maintenance, ER to Golgi transport, transport within the Golgi, and two components of the ESCRT III complex associated with sorting of cargo proteins. As explained for nuclear transport, protein synthesis, and mRNA decay, vesicle transport is a process not known to be directly involved in retrotransposon integration. However, vesicle traffic and membranes are critical for the replication of many viruses. Examples include gamma and Type-D retroviruses, both of which require the endosomal system to traffic Gag and Gag-Pol to the plasma membrane [63]. This contribution to the replication of retroviruses occurs much earlier in the lifecycle than integration, the stage of Tf1 activity that requires vesicle transport.

Nine (15%) of the candidate integration factors identified in our screen are associated with vesicle transport. Several vesicle formation, cargo loading, and vesicle transport factors are involved in Ty1 retrotransposition including functions that occur after cDNA is synthesized (Table 2). Interestingly, a particularly large set of vesicle trafficking factors contribute to Ty3 retrotransposition post reverse transcription [20]. These include several components of ESCRT complexes I, II, and III (Snf7, Vps4, Vph1, Vps20, Bro1, Vps28, Snf8, Vps36, Clc1, Fab1, and Vma7). The high numbers of vesicle trafficking factors involved in Ty1 and Ty3 transposition indicate late stages of LTR-retrotransposition require highly conserved features of vesicle trafficking.

Four Tf1 candidate integration factors function in ubiquitination, deubiquitination, or assembly of the proteasome (Table 1). These factors contribute to the degradation of a wide range of proteins, any number of which could be important for integration. It is therefore difficult to propose specific functions of these candidates that promote integration. With such broad impact on cellular systems, it’s not surprising that ubiquitin modifications and the proteasome factors promote activities of Ty1 and Ty3 [18–21].

A set of eight metabolic enzymes was identified in the list of candidate integration factors (Table 1). They are mostly unrelated making it difficult to identify a specific pathway that might mediate integration. The exception is that the metabolic factors included both enzymes responsible for synthesis of trehalose, a disaccharide that mitigates the impact of heat and oxidative stress [64–66]. What is more intriguing is that one of these enzymes trehalose-phosphate synthase (Tps2) is important for Ty1 transposition [22]. Although with Ty1, Tps2 is required for an early step in the transposition cycle that is necessary for cDNA production.

### Chromatin associated host factors that promote integration

Candidate integration factors that are chromatin associated included the histone variant H2A.Z (Table 1). H2A.Z is concentrated in the +1 and -1 nucleosomes that flank the nucleosome depleted region of promoters [67, 68]. Genome-wide profiles of 1.6 million insertions show that Tf1 targets the nucleosome-depleted region of promoters in a window of 150 bp immediately adjacent to the -1 and +1 nucleosomes [32]. With this pattern of integration, it is feasible that H2A.Z participates in integration via a direct interaction with IN. Alternatively, it is possible that H2A.Z recruits the binding of a targeting factor or contributes to a form of chromatin structure that facilitates efficient integration. Nucleosomes are determinants of integration for retroviruses due to structural perturbations of the DNA [69–71]. Interestingly, H2A.Z and the remodeling factor that assembles H2A.Z in nucleosomes, Swr1, are important for Ty1 transposition [18, 19]. Importantly, H2A.Z and Swr1 may function directly in Ty1 integration since deletion of *swr1* does not reduce Ty1 cDNA and H2A.Z associates with RNA pol III promoters [18, 72, 73]. A role of H2A.Z in integration is consistent with the strong association observed between H2A.Z and sites of Ty1 integration [74]. For a factor to promote the integration of two highly divergent LTR-retrotransposons such as Tf1 and Ty1 suggests that H2A.Z contributes to a feature of chromatin structure that is important for the integration of a broad range of LTR-retrotransposons.

Candidate integration factors associated with chromatin included components of histone modifying complexes (Set1, Nts1, and Ubp8) and Snf5, a subunit of the SWI/SNF chromatin-remodeling complex (Table 1). A number of factors with similar functions contribute to Ty1 and Ty3 transposition, possibly in integration (Table 2). Set1 is the histone H3 lysine 4 methylase component of the COMPASS complex. A different component of this complex, Swd1, contributes to Ty3 transposition post-reverse transcription [21]. Nts1 is a component of the histone H3 deacetylase complex Clr6 and Ubp8 is a subunit of the SAGA histone acetylation complex. A number of factors controlling histone acetylation promote transposition in *S*. *cerevisiae* (Table 2). The SWI/SNF complex has global impact on gene regulation including Ty1 transcription [75]. As a result, Snf5 and other components of SWI/SNF contribute to Ty1 transposition. Although chromatin modifications and remodeling have broad effects on expression of the genome, the similarities in the chromatin complexes that promote transposition of Tf1, Ty1, and Ty3 suggest certain features of chromatin structure may play a common role in integration of LTR-retrotransposons.

Another class of candidate integration factors we identified is associated with transcription (Table 1). Ckb1 is a regulatory subunit of casein kinase 2 and Lkh1 is a kinase. Both factors mediate the phosphorylation of a broad range of substrates including transcription factors, and subunits of RNA polymerases [76–79]. Srb11 is a cyclin-like component of RNA polymerase II involved in phosphorylation of the RNA polymerase II C-terminal domain [80, 81]. Any of these kinase functions have the potential to modulate a protein important for integration. Paf1, and Tfs1 associate directly with RNA pol II and have the potential to target integration directly. Interestingly, deletion of *paf1* abolishes the methylation of histone H3K4. Paf1 controls H3K4 methylation by promoting ubiquitylation of histone H2B, which is required to recruit Set1, [82] a factor our screen identified. This connection suggests that the role of Paf1 in integration is to promote H3K4 methylation. Interestingly, the Paf complex and rad6 inhibit integration of Ty1 and prevent disruption of ORFs [23, 74, 83–85]. Genome-wide integration of Ty1 upstream of pol III genes does not change in a *rad6* deletion [74]. Proposed models for these observations suggest Paf and Rad6 strengthen target specificity and restrict integration. These effects are mechanistically distinct from the contribution Set1 and Paf1 make to Tf1 integration.

Two core splicing factors, Cwf12, and Smd3, and the splicing coactivator Pwi1 were identified in our screen as candidate integration factors. Although it’s possible that splicing factors were identified because the transposition assay relies on splicing of the artificial intron, this is unlikely because the intron must also be spliced for cDNA recombination to be detected. Cwf12 is a member of the NineTeen Complex that plays a central role in splicing by tethering the U6 snRNA to the activated spliceosome [86–89]. Smd3 is one of seven Sm proteins that are common components of the U1, U2, U4, and U5 snRNPs [86]. It is not clear whether these splicing components directly contribute to integration as it is possible their absence changed expression of proteins that mediate integration. However, several core splicing complexes including the NineTeen Complex contribute to stages of Ty1 transposition after reverse transcription and the splicing regulator Sqs1 promotes stages of Ty1 and Ty3 transposition post reverse transcription (Table 2). While there is no information about how splicing could contribute to integration in yeast, recent studies of HIV-1 found that the host factor LEDGF/p75 interacts with splicing factors and targets integration to highly spliced genes [90, 91].

### The contribution of DNA repair factors to integration

Our genetic screen found deletion of genes encoding four DNA repair factors, Rhp18, Rhp23, Rad50, and Rad51 resulted in significant reductions in transposition without lowering homologous recombination or expression of Gag and IN (Table 1). If these factors mediate integration it is possible they function with the targeting factor Sap1 which can be a replication fork barrier [92]. Rad50 and Rad51 mediate homologous recombination and this activity can contribute to DNA replication by assisting recovery of arrested replication forks [93]. As a result, it’s possible that Rad50 and Rad51 interact with Sap1 at arrested forks in a configuration that stimulates integration. This is consistent with the model that Sap1 induces Tf1 integration at stalled forks [33].

However, the functions of the DNA repair factors in Table 1 are broad suggesting the intriguing possibility that these factors are responsible for repairing the unattached 5’ ends of the integrated cDNAs. The integrases of LTR-retrotransposons and retroviruses catalyze DNA-strand transfer reactions where the 3’ ends of the cDNAs attack staggered phosphodiester bonds on opposite strands of the target DNA [94, 95]. The inserts are flanked by single stranded gaps with 5’ ends of the cDNA unattached to the target site. These gaps must be repaired and this process is of great interest as it is unknown which factors are responsible for integration repair of any LTR-retrotransposon or retrovirus. Deletion strains unable to repair the single stranded DNA gaps would have reduced transposition activity but potentially maintain normal frequencies of homologous recombination.

Rhp18 is the *S*. *pombe* homolog of Rad18, an E3 ubiquitin ligase that binds single stranded DNA and functions both in postreplication repair and in translesion synthesis [96–100]. Additional evidence indicates that Rad18 in mammalian cells mediates homologous recombination repair of double-strand breaks [101]. Rad18 localizes to double-strand breaks and facilitates homologous recombination by interacting directly with Rad51, a RecA family recombinase. Rad51 was also identified as a candidate integration factor suggesting that Rad18 and Rad51 could function together in homologous recombination to repair integration sites (Table 1). If replication occurs before the single strand gaps are repaired, then the resulting double strand breaks could be repaired by homologous recombination.

Rad50, another DNA repair factor that promoted Tf1 integration (Table 1), is a subunit of Mre11-Rad50-Xrs2 MRX complex responsible for resection of double-strand breaks [102]. This function not only contributes to homologous recombination but is also thought to be important for processing unusual DNA structures. One possibility is that MRX is important for repairing integration sites because it displaces IN. Studies of Mu phage show that the transpososome adheres tightly to integration sites and is removed by the ClpX protease [103, 104].

Rhp23, the *S*. *pombe* homolog of Rad23 is another DNA repair factor found to promote integration (Table 1). Rhp23 is a subunit of Nuclear Excision Repair Factor 2 with Rad4p that binds damaged DNA and excises fragments of 24 to 27 nucleotides [105]. One other candidate integration factor with the potential to repair DNA is Mhf2, discussed above as a component of kinetochores (Table 1). Mhf2 is a component of the MHF histone-fold complex that in human cells interacts with both DNA and the Fanconia anemia associated factor FANCM to repair damaged DNA and stabilize replication forks stalled by DNA interstrand crosslinks [106]. This function may participate with Rhp18 in conducting translesion synthesis.

In all, five candidate integration factors identified with our screen, Rhp18, Rhp23, Rad50, Rad51, and Mhf2 have DNA repair activity and therefore have the potential to repair integration sites. They participate in translesion synthesis (Rhp18 and Mhf2), double strand break repair (Rhp18, Rad51, and Rad50), and nuclear excision repair (Rhp23). It is possible these factors function in concurrent repair processes that serve redundant functions. It is also possible that there are other factors important for repairing integration sites that were not identified by our screen because they contribute to homologous recombination of cDNA. These would be factors such as Rad52 that are important for both homologous recombination and transposition (S2 Table).

It is significant that similar DNA repair factors are involved in retrotransposition in *S*. *cerevisiae* (Table 2). In particular a subunit of the MRX complex (Xrs2) and a Rad51 mediator (Rad52) contribute to Ty1 transposition [22]. Consistent with a role in integration site repair, the contribution of these DNA repair factors occurs after cDNA synthesis. Several other studies independently found members of the MRX complex and the Rad51-Rad52 recombination pathway are involved in Ty1 transposition [23, 24, 107–110]. However in these studies the DNA repair factors inhibit transposition as measured with a single copy Ty1 carrying the *his3*AI reporter. Amounts of Ty1 cDNA produced by single copy Ty1 increase in the absence of Rad51-52 factors. The dramatic increase in cDNA in these assays is triggered by DNA damage and requires S-phase checkpoint factors [111]. It is not clear why single copy Ty1 with the *his3AI* reporter produces such differences from Ty1 and Tf1 expressed from a plasmid. Nevertheless, the overlap of DNA repair factors that can promote Ty1 and Tf1 transposition argues these factors may mediate a conserved feature of integration.

In a previously published study designed to identify factors that repair DNA at HIV-1 integration sites, 232 genes associated with DNA repair were tested with RNAi methods [112]. A cluster of six genes involved in short patch base excision repair were identified that when deleted in mouse embryo fibroblasts resulted in decreased HIV-1 replication. The proteins identified included damage recognition glycosylases (OGG1 and MYH) and the late repair factor POLβ. Consistent with a role in integration site repair these proteins promote late steps in replication that occur after reverse transcription and nuclear entry [113]. While these proteins as well as the candidate integration factors we identified may be involved in the repair of integration sites, further studies are needed that can directly test this model.

The overlap of factors and pathways that promote late stages of retrotransposition in *S*. *cerevisiae* and *S*. *pombe* suggest these represent cellular processes that are fundamental to delivery of cDNA or integration. The list of genes identified in screens of *S*. *cerevisiae* and *S*. *pombe* in Table 2 shows a number of overlapping pathways but it is not a formal test that can be evaluated statistically. To address this, we assembled lists of genes important for Ty1 and Ty3 transposition that when mutated do not result in significant reduction of cDNA. The genes of *S*. *cerevisiae* along with those we identified from *S*. *pombe* were grouped by gene ontology using Fission Yeast gene ontology slim terms (S4 Table). We calculated the enrichment of these genes in each slim term relative to the total number of non-essential genes in the slim term that are included in the deletion sets (S7 Fig). Although the overall number of genes identified by these genetic screens are relatively low to calculate enrichment values for non-essential genes, several had enrichments with p values <0.05 (S7 Fig). The slim terms that showed statistically significant enrichment for genes important for late stages of transposition in *S*. *pombe* and *S*. *cerevisiae* were RNA metabolic processes and protein catabolic processes. While not reaching p values <0.05, other terms showed enrichment near two-fold for late stage transposition genes of *S*. *pombe* and *S*. *cerevisiae* such as cell adhesion, chromatin organization, nucleocytoplasmic transport, regulation of transcription, DNA repair, and protein targeting. While the slim terms are broader than what we described in Table 2, they do reflect the overlap between functions implicated in late stages of transposition in both *S*. *pombe* and *S*. *cerevisiae*.

### The role of candidate integration factors in target site selection

The 61 factors that promote integration participate in a wide range of cellular processes. We sought additional evidence about whether these processes are directly involved in integration by testing a representative set of candidate integration factors for contributions to cDNA levels and insertion site distribution. We evaluated strains lacking DNA repair factors (Rad50 and Rad51), chromatin factors (Pht1 and Set1), the chromatin remodeler Snf5, the nuclear pore protein Nup61, and the splicing factor Cwf12. Although our recombination assays indicated cells lacking these candidates had wild-type levels of cDNA in the nucleus (S3 Table), it was possible that incomplete cDNAs or intermediates were responsible for the recombination. We used a DNA blot to detect altered structure and accumulation of the Tf1-*nat*AI cDNA. The cDNA produced from the plasmid expressed Tf1-*nat*AI was digested with BsrGI and quantified on DNA blots (S8 Fig). The 2.9 kb band detected with a probe for *nat*AI is produced by BsrGI cleavage of the 3’ section of the cDNA. This terminal double stranded portion of cDNA is synthesized only after minus and plus strand transfers and as a result is a measure of mature Tf1-*nat*AI cDNA. The intensities of the cDNA bands were quantified and normalized relative to the amount of expression plasmid in each strain. No reduction in cDNA was observed in cells lacking Rad50, Rad51, Pht1, Set1, Snf5, Nup61, or Cwf12. Interestingly, cDNA was elevated in cells lacking Rad51 and was modestly increased in the absence of Pht1, Snf5, and Nup61.

**Fig 5:**
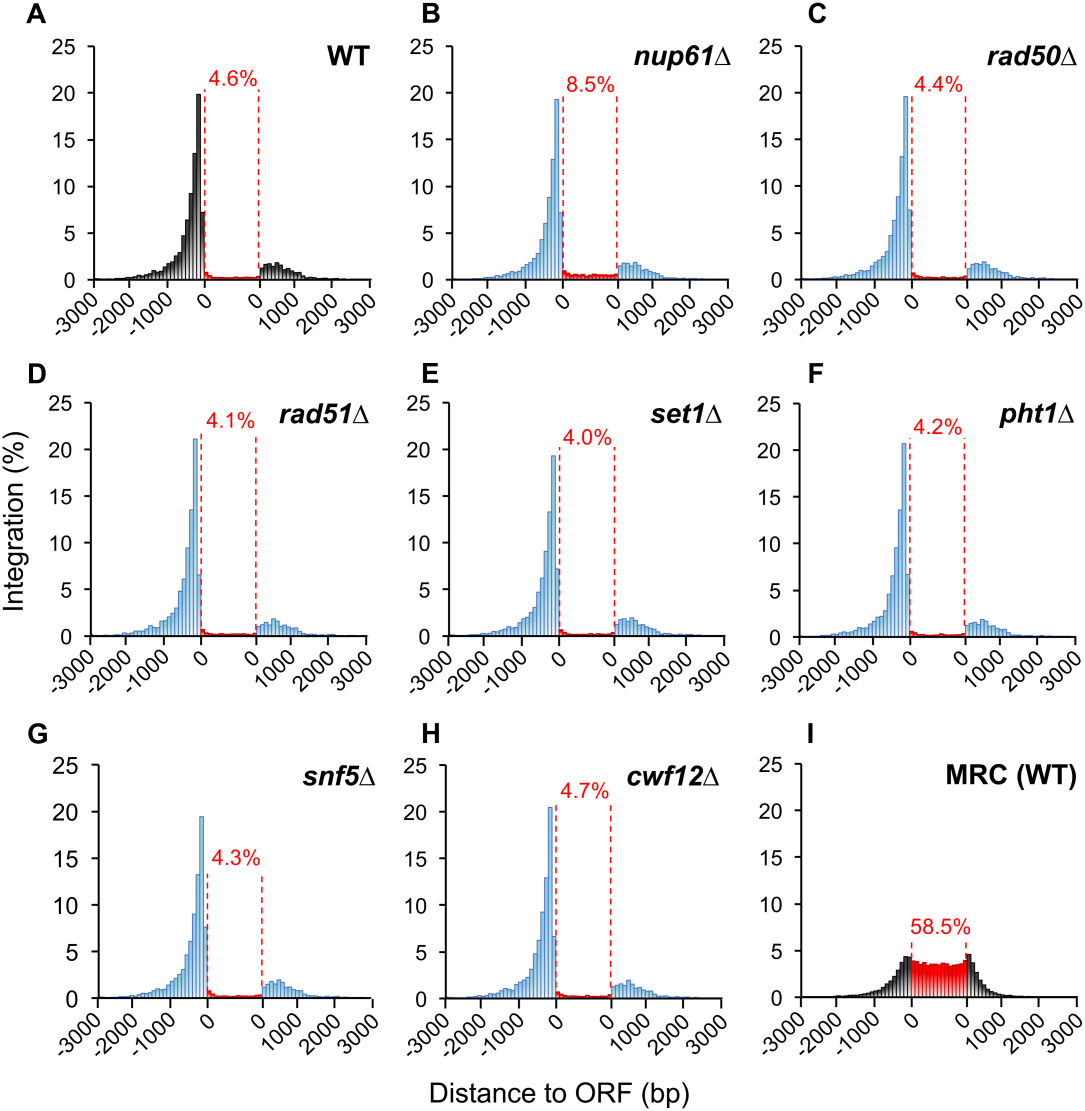
Tf1 integration clustered upstream of ORFs in both wild-type and the strains with transposition defects. The x-axis is the distance upstream (-3000 bp to 0 bp) and downstream (0 bp to +3000 bp) from ORFs divided into bins of 100 bp. The y-axis is the amount of integrations within a bin as a percent of all integrations. Insertions closer to the 5’ end (-) of an ORF were plotted upstream of the ORF and insertions closer to the 3’ end (+) were plotted downstream of the ORF. The red vertical dashes delineate the body of ORFs, and insertions in ORFs are tabulated within 15 bins of equal proportion; total insertions in ORFs are labeled in percentages. (A) Wild-type (indicated with black bars); (B-H) deletion mutants (indicated with blue bars); (I) a matched random control of integrations in wild-type cells.

We determined whether these seven representative candidates contributed to integration site distribution by high throughput sequencing inserts produced by plasmid-derived expression of Tf1-*nat*AI (Materials and methods). We used the Illumina platform and sequenced ligation-mediated PCR libraries of integration sites (S5 Table) [30–32]. We quantified integration in ORFs divided into 15 equal segments. For insertions upstream and downstream of ORFs we summed them in 100 bp windows (Fig 5). As observed in previous studies, integration clustered upstream of ORFs (Fig 5A) [30–32, 114, 115]. Although all the deletion strains tested exhibited this clustering upstream of ORFs, deletion of *nup61* resulted in a modest increase of integration within ORFs (Fig 5B). When integration sites are selected at random in the Matched Random Control (MRC) 58.55% occurred within ORFs (Fig 5I).

**Fig 6:**
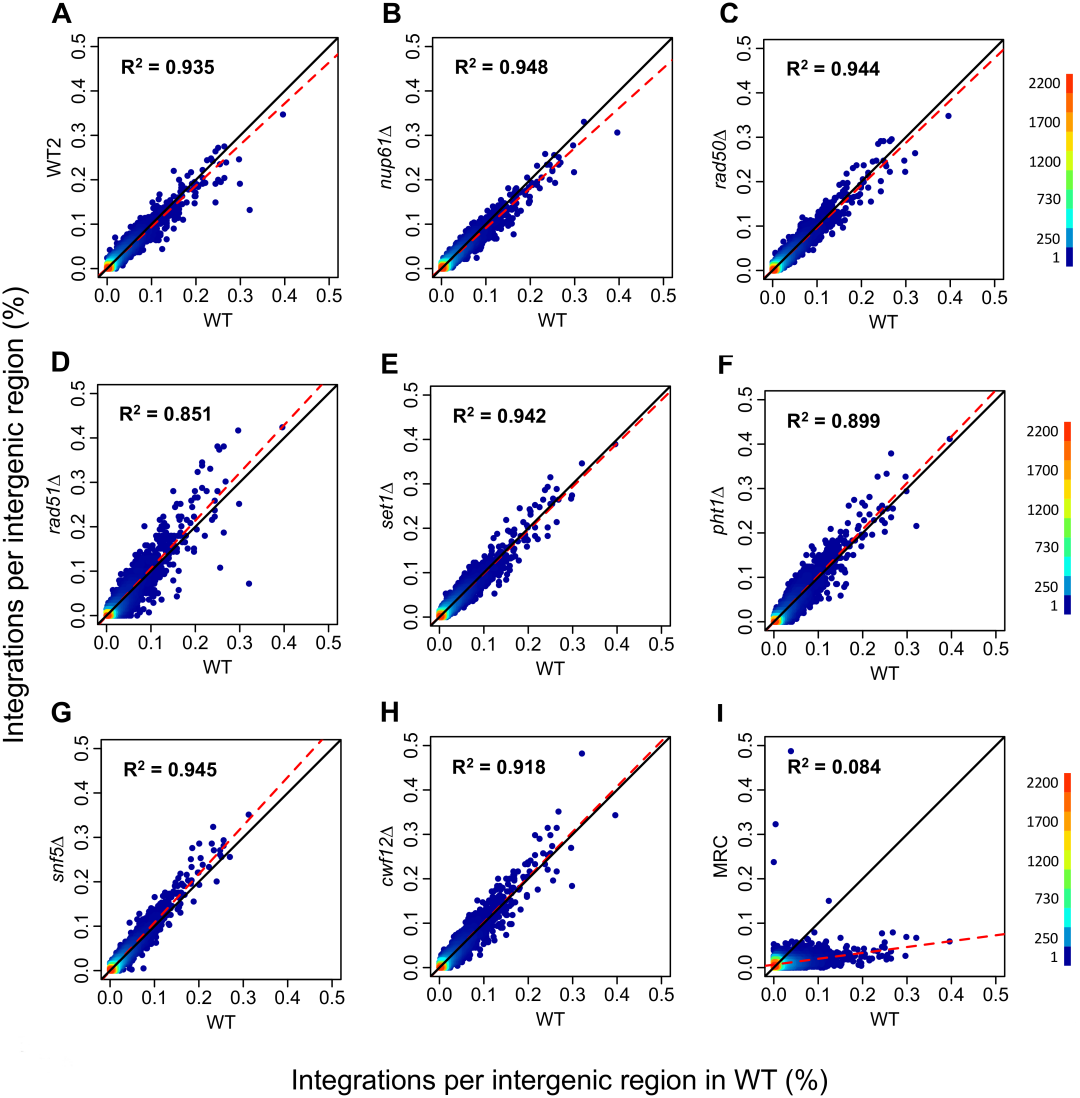
Distribution of Tf1 integration within intergenic regions in transposition-defective mutants compared to wild-type. Density scatter plot and linear regression analysis are shown for each indicated deletion strain. The x-axis is the amount of insertions in WT cells per intergenic region normalized as a percent of all insertions and sorted by increasing amount of integrations. The y-axis is the corresponding normalized insertion number per intergenic region in the deletion (Δ) mutant. Data points are plotted such that color gradient indicates the density of overlapping points. The correlation coefficient (R^2^) from linear regression of each WT/Δ pair is indicated and a trend line is shown in red dash. A diagonal reference line (y = x) is shown in black. A. WT plotted against a biologically independent set of integration in wild-type cells, WT2. B-H, WT plotted against deletion mutants, I, WT plotted against MRC.

Reproducible measures show integration levels in intergenic sequences vary over a wide range with the bulk of insertions occurring in 1,000 of the 5,000 intergenic regions in the genome [30–32]. We asked whether this subgroup of candidate integration factors contribute to the distribution of integration among intergenic regions. When comparing amounts of integration in intergenic regions, strains lacking the candidate integration factors had strong correlations with the wild-type strain (Fig 6). These correlations were comparable to what we observed between two independent experiments with integration sites produced by wild-type cells (Fig 6A). This indicates that these factors did not significantly contribute to the targeting of integration in intergenic regions.

**Fig 7:**
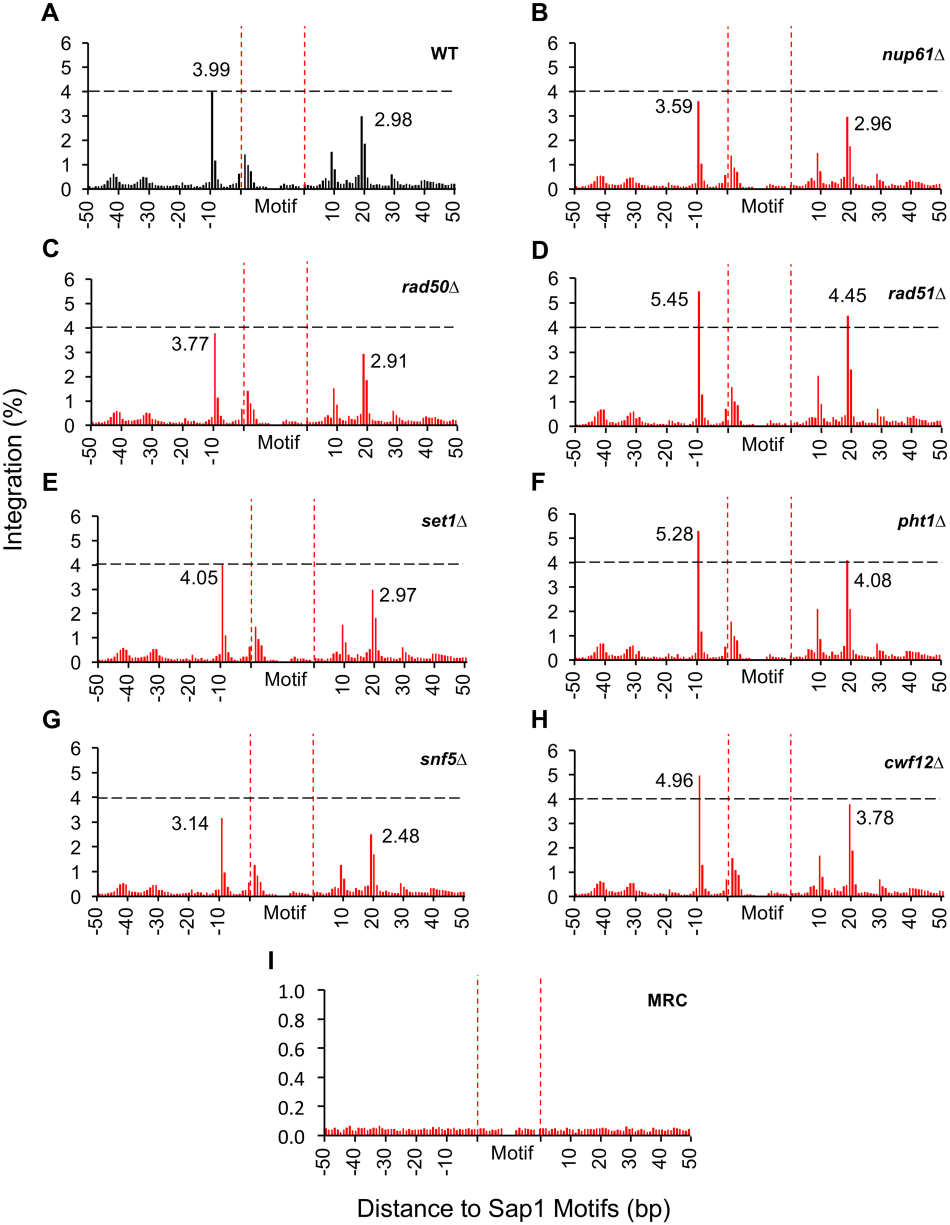
Targeting specificity of Tf1 at Sap1-binding motifs is retained in transposition defective mutants. Insertions were tabulated at single nucleotide positions relative to 5000 Sap1-binding motifs in the *S*. *pombe* genome [32]. The x-axis is the distance (bp) upstream (-) and downstream (+) of the 21-bp motif measured for 50 bp in each direction. The y-axis is the amount of integrations at each nucleotide position as a percent of all integrations. The individual strains analyzed were A. Wild-type, B-H, deletion mutations, I, MRC.

Our study of Tf1 integration showed that insertions cluster adjacent to positions of Sap1 binding at bases -9 and +19 relative to the motif recognized by Sap1 [32]. We asked whether the residual integration in the representative set of deletion mutations occurred at the -9 and +19 positions relative to the 5,000 best matches to the Sap1 motif. The integration pattern relative to the Sap1 motif was largely unchanged in the deletions (Fig 7). These patterns suggest the residual integration in the deletion mutations retains its dependence on Sap1.

### Rhp18 and Cwf3 physically interact with IN

**Fig 8:**
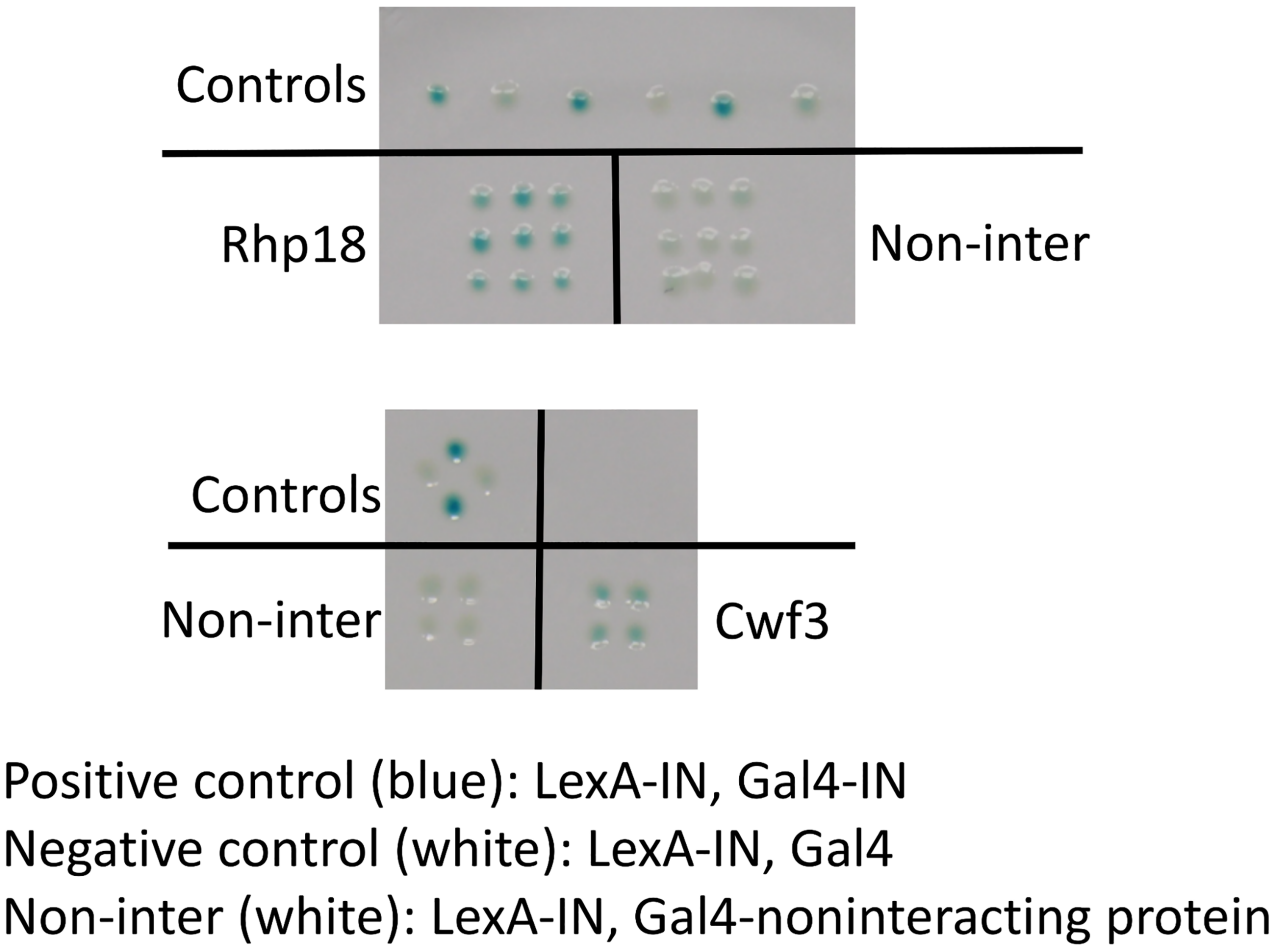
Interactions of IN with Rhp18 and Cwf3 as detected by two-hybrid assays. Interactions between IN and IN, Rhp18, and Cwf3 resulted in lacZ expression that was detected as blue CTY10-5d cells on nitrocellulose filters. The multimerization of IN produced by LexA-IN and Gal4-IN was our positive control. Technical replicates of this positive control produced the three blue patches on the top panel and the two blue patches on the bottom panel. The negative control was cells expressing LexA-IN and Gal4. Technical replicates of this negative control produced the three white patches on the top panel and the two white patches on the bottom panel. Another negative control was cells expressing LexA-IN and Gal4 fused to a non-interacting protein. Nine independent transformants expressing LexA-IN and Gal4-Rhp18 produced blue coloration indicating a significant interaction. Four independent transformants expressing LexA-IN and Gal4-Cwf3 also produced blue signal indicating interaction.

Candidate integration factors that contribute directly to integration may interact physically with IN. In a report to be published separately, we applied the two-hybrid system of *S*. *cerevisiae* to identify host factors that interact with Tf1 IN. We found that the DNA repair factor Rhp18 reproducibly interacts with IN (Fig 8). Rhp18 was one of the candidate integration factors (Table 1), indicating that our screen was able to identify factors directly involved in integration. The interaction of a DNA repair factor with IN is intriguing and suggests the possibility that Tf1 recruits repair factors to integration sites to facilitate repair. This IN mediated recruitment may be a conserved function of integration since the human homolog of Rhp18, hRad18 interacts and co-localizes with HIV-1 IN in HEK293T cells [116].

Our two-hybrid survey also identified an interaction between the Cwf3 component of the NineTeen splicing complex and Tf1 IN (Fig 8). One of the candidate integration factors, Cwf12, is also a member of the NineTeen complex indicating that the NineTeen complex is directly involved in integration [88, 89]. A role of splicing has been observed for HIV-1 where integration is directed to genes that are highly spliced [90]. Perhaps the NineTeen complex plays a similar role in *S*. *pombe* by recruiting Tf1 IN to sites of integration. The two-hybrid interactions described above resulted from a screen of a cDNA library. Since such screens are not exhaustive it is possible and even likely that IN interacts directly with other candidate integration factors listed in Table 1.

### Conclusions

Our screen of 3,004 non-essential genes represents the first comprehensive study of host factors in *S*. *pombe* that promote retrotransposition. With our combination of genetic assays we were able to identify factors that may contribute directly to integration. However, other experiments are needed to evaluate the candidates for a direct role in integration. In addition to promoting integration some of our candidates could mediate a different step late in transposition such as the localization of cDNA in a nuclear compartment. Our data makes it possible to compare the candidate integration factors we identified in *S*. *pombe* to the factors of *S*. *cerevisiae* that through a number of studies are likely to promote integration. Factors we identified function in nuclear transport, protein synthesis, mRNA processing, vesicle transport, chromatin structure, transcription, spicing, and DNA repair. Although this wide range of host factors suggests many could make indirect contributions to integration, we found a surprising overlap with pathways and factors important for integration of Ty1 and Ty3 in *S*. *cerevisiae* (Table 2). These overlaps support the model that many of these processes contribute directly to integration. The extent of overlap is significant because of the great evolutionary distance between these yeasts and because Ty1 belongs to the copia family, a distinct superfamily of LTR-retrotransposons from gypsy, the family that includes Tf1 and Ty3. The consensus this study provides serves as an opportunity to design experiments that test these pathways for mechanisms that drive integration of retroviruses in humans. Our data also provide an important first view of factors that may repair integrated DNA. We expect there are other factors that repair integrated DNA that we did not identify because they also contribute to cDNA recombination. To ask whether repair of integration is broadly conserved, assays will be needed that detect integrated cDNA with unrepaired 5’ ends. These experiments will be able to measure the contribution of each factor to the repair of integrated cDNA.

## Materials and methods

### Media

Edinburgh Minimal Medium (EMM) was prepared as described [117]. PM was identical to EMM except the nitrogen source was 3.74 gm/l monosodium glutamate. Minimal media were supplemented with 2 gm/l of a dropout mixture that contained equal weights of all amino acids and adenine was added to 2.5 times the weight of the other components [29]. When indicated vitamin B1 was added to a final concentration of μM and 5-Fluoroorotic acid (FOA) (U.S. Biologicals, Swampscott, MA.) was added to a final concentration of 1 mg/ml. When FOA is used in EMM the final concentration of uracil is lowered to 50 μg/ml. The rich medium, yeast extract plus supplements (YES) contained 5 g/l Difco yeast extract, 30 g/l glucose, and 2 g/l dropout powder. When indicated the drug nourseothricin (Nat), (ClonNAT, Jena Bioscience, Germany) was added to a final concentration of 100μg/ml.

### Plasmid construction

The plasmids for this study are listed in S6 Table. The plasmid pHL2882, used to measure transposition in the deletion strains, includes the *nmt1* promoter to express Tf1 with *nat* disrupted with an artificial intron (*nat*AI) (S1 Fig and Fig 1A). pHL2883 and pHL2884 were equivalent to pHL2882 except they have frame shift mutations in PR and IN, respectively. These plasmids were derived from pHL2803, which expressed Tf1 with a *nat* marker that lacks the AI. pHL2803 was constructed starting with pHL2673 by replacing the BsrGI-BamHI fragment containing IN sequence and *neo* with a BsrGI-BamHI fragment that was generated by fusion PCR to introduce restriction sites for AsiSI, SacII, and NotI just upstream of the polypurine tract. The primers for this fusion PCR and all other oligonucleotides are listed in S6 Table. To complete pHL2803, *nat* was PCR amplified with primers containing AsiSI and NotI restriction sites and the product was inserted with *nat* in reverse orientation to Tf1 into pHL2673 with the AsiSI and NotI sites. To produce pHL2804 (PRfs) and pHL2805 (INfs), the AvrII-BsrGI fragments of Tf1 from pHL415-2 (PRfs) and pHL431-25 (INfs) were inserted into the AvrII-BsrGI backbone of pHL2803. pHL2882 was generated by inserting *nat*AI synthesized commercially by DNA 2.0 into the AsiSI and NotI sites of pHL2803 (S1 Fig). The synthetic fragment contained the AI located after the 60^th^ amino acid of Nat (S1 Fig). The codon usage of the *nat* ORF was optimized for *S*. *pombe* without changing the amino acid sequence. pHL2883 and pHL2884 were created by inserting the BsrGI-BamHI fragment with *nat*AI from pHL2882 into the backbones of pHL2804 and pHL2805, respectively.

pHL2898, pHL2900, and pHL2902 express Tf1-*neo*AI from the *nmt1* promoter and encode the IN mutations D987N, D1047N, and E1083Q, respectively. These plasmids were made by replacing the BsrGI-NarI fragment of pHL449-1 with PCR fusion products of the BsrGI-NarI fragment containing the mutations. The primers for these PCRs are listed in S7 Table.

### *S*. *pombe* strains

The deletion library contained 3,004 haploid deletion strains from the V2 library of Bioneer (Alameda, CA, Cat. # M2030) [34]. The deletions were derived from two haploid parents ED666 (*h+ ade6-M210 ura4-D18 leu1-32*) and ED668 (*h+ ade6-M216 ura4-D18 leu1-32*). These strains and others are listed in S8 Table.

### Introduction of Tf1-*nat*AI expression plasmid pHL2882 in the deletion library

To transform pHL2882 in all 3,004 deletion strains, we modified previously published protocols [118]. Using a sterile 96 pin multi-replicator (Model-VP408FS2AS-1, V&P Scientific, Inc, San Diego, California,USA), each 96 well plate of the library was pined onto single well YES agar plates, and incubated at 32°C for 72hrs (Fig 2A). Each strain was inoculated with an initial OD_600nm_ of 0.05 units in 5ml YES liquid media in 15ml tubes. All 96 deletion strains from each plate were independently transformed with pHL2882 (10μg) and 5μg of sonicated herring sperm DNA. Half of each culture was transformed with herring sperm DNA and no pHL2882 as a control for contamination. The transformed cells were processed as indicated in Fig 2A. We isolated four independent transformants for each deletion strain.

### Transposition assay

Strains containing Tf1-*nat*AI (pHL2882) were grown as patches on agar plates with PM-U+L+B1. These patches were then replica printed on to agar plates with PM-U+L-B1 to induce the *nmt1* promoter. After 4 days of incubation, the patches were replica printed onto agar plates with EMM+U+L+B1+FOA twice in succession, the first print was incubated 3 days and the second for 2 days. The patches were then replica printed on to YES+Nat+FOA agar and incubated for 44hrs at 32°C (Fig 3). Each transposition plate contained patches of PRfs and INfs as controls and the growth of the deletion strains was scored relative to a set of standards (S2 Fig). Four independent transformants of each deletion strain were assayed. An average transposition score was determined if all four transformants had scores within a window of three units. Outliers were excluded from the average if a single transformant had a difference in score three units or greater from the other three. If two transformants had scores that differed by three or more units from the other transformants, the score for the deletion was considered to be inconsistent and were excluded from the screen.

### Homologous recombination assays

To measure amounts of Tf1-cDNA in the nucleus, we used a homologous recombination patch assay as described [29, 36]. Deletion strains containing pHL2882 were grown on PM-U+L+B1 agar plates for 3 days at 32°C. The patches were replica printed onto PM-U+L-B1 for induction. After 4 days of incubation, the patches were replica printed onto YES+Nat agar and incubated for 24hr at 32°C. The patches were compared with the PRfs and INfs controls from the same plate and scored for homologous recombination using standards (S3 Fig). Four independent transformants of each deletion strain were assayed. The adjusted average scores were determined as previously described in the transposition assay.

Strains tested with the quantitative homologous recombination assay were grown on PM-U+L+B1 plates for 3 days at 32°C (S4 Fig). Cells were then suspended into 5ml of PM-U+L-B1 liquid media, and washed six times with 5 ml of PM-U+L-B1 liquid media to remove residual B1. Cells were then inoculated in 5ml of PM-U+L-B1 media at a starting OD_600nm_ of 0.05 units. Following 4 days of incubation the cultures were diluted to OD_600nm_ 1.0 (2x10^7^ cells/ml) in PM-U+L+B1 medium and serially diluted from 2x10^7^ cells/ml to 2 x10^4^ cells/ml using PM-U+L+B1, then spread on YES and YES+Nat (100 μg/ml) agar plates and grown for 3 days at 32°C. Colonies were counted per plate, and the homologous recombination frequencies were determined with the following equation:

$$Quantitative\;Homologous\;Recombination\;Frequency\;=\;\frac{(number\;of\;colonies\;on\;YES+NAT)\;*\;100}{\left(number\;of\;colonies\;on\;YES\;*\;dilution\;factor\right)}$$

Recombination frequencies for wild-type Tf1-*nat*AI in wild-type strains without deletions ranged from 3% to 1.5% in individual experiments. Values for each deletion strain were normalized to wild-type strains assayed during the same experiment.

### Immunoblots

10 ml cultures were inoculated with a starting OD_600nm_ of 0.05 units. After 18 hours, cells were washed with sterile deionized water. The cell pellets were suspended in 0.4ml of extraction buffer consisting of 15 mM KCl, 10 mM HEPES-KOH (pH 7.8), 5 mM EDTA, 5 mM dithiothreitol, protease inhibitor cocktail tablet (Complete, Roche Lifesciences), 2 mM phenylmethylsulfonylfluoride (PMSF), Pepstatin(0.7mg/ml, 1000x stock), leupeptin (0.5mg/ml 1000x stock), and Aprotinin (1.0mg/ml 1000x stock). An equal volume of acid-washed glass beads was added and vortexed in a bead beater for a total of 3 min in 30 sec intervals separated by 30 sec rest. 0.1 ml of extraction buffer was mixed into the extract, and the liquid was removed. Extracts were combined with 2X sample buffer and boiled. The samples were loaded onto an SDS–10% polyacrylamide gel. The gels were electrotransferred to Immobilon-FL membranes (Millipore). The production bleeds of 660 (anti-Gag) and 657(anti-IN) were used to probe Tf1-IN and Gag protein levels, and monoclonal anti-α-Tubulin antibody (Sigma-Aldrich,USA) was used as a loading control on all immunoblot experiments. The anti-alpha tubulin, 660 (anti-Gag) and 657(anti-IN) were used with 1:5000, 1:10,000, and 1:5000, respectively. The fluorescently-tagged secondary mouse IR-Dye 700 and rabbit anti-body IR-Dye 800 (Rockland Immunochemicals Inc.Limerick, PA) were used with 1:20,000 dilutions. The Immobilon-FL membranes were scanned with an Odyssey infrared imaging system (LI-COR Biosciences). Fluorescence levels from antibodies specific for Gag and IN were normalized to amounts of tubulin and measured with a Li-COR digital instrument (Materials and methods) (S3 Table).

For the deletion strains tested two independent transformants were assayed for Tf1-IN and Gag protein levels. Geometric means of Gag and IN levels of these replica pairs of deletion strains were compared to the geometric means of the wild-type strains lacking the deletion.

The Tf1-IN and Gag protein expression levels were measured and normalized to alpha tubulin. The fold change in Tf1-IN and Gag protein expression were calculated using below equation:

$$Change\;in\;IN\;and\;Gag\;levels\;=\;\frac{Normalized\;geometric\;mean\;of\;IN\;and\;Gag\;protein\;in\;mutant}{Normalized\;geometirc\;mean\;of\;IN\;and\;Gag\;in\;wild\;type\;strain}$$

### Quantification of cDNA by DNA blot

Tf1 was expressed by incubating the cells for 2 days in 50 ml of EMM –B1 starting at OD600 = 0.05 to induce the nmt1 promoter after washing them 4 times in EMM –B1. Genomic DNAs were isolated from 200 OD units of the resulting cultures. Southern blots were performed as described previously [36, 47] with the following modifications. The nat probe was produced by digesting 5μg of pHL2597 with 160 units of EcoRI, isolating the 1.2 kb fragment from a 0.7% agarose gel and random-priming labelling with ^32^P-CTP. One microgram of gDNAs were digested with 40 units of BsrGI, separated on a 1.0% agarose gel and transferred to a nylon membrane. The blot was hybridized with the nat probe. BsrGI digestion resulted in Tf1 cDNAs being detected at 2.8 kb and the Tf1 expression plasmid at 14kb. Tf1 cDNA was quantified with phosphoimaging and normalized to the amount of expression plasmid. Briefly, the ^32^P-signal was detected by phosphoimaging on a Typhoon FLA-9500. The relative level of cDNA was determined by normalizing the signal intensity of the 2.9kb cDNA band to the signal intensity of the 14kb plasmid band.

### High throughput sequencing of Tf1 integration

Tf1 transposition was induced in strains containing Tf1-natAI (pHL2882) and deleted for *pht1*, *rad51*, *set1*, *cwf12*, *snf5*, *nup61*, *rad50* or wild-type (S8 Table) as described previously but with some modifications [31]. Briefly, cells were washed 4 times in EMM –B1 before being inoculated at OD600 = 0.05 in EMM –B1 to induce the *nmt1* promoter, then grown 4 days for each of two passages. Cells with transposition events were selected by incubating them in 50 ml of EMM+B1+FOA for 4 days followed by 4 days in YES+FOA+Nat. Genomic DNAs were isolated from 200 OD units of the resulting cultures. Libraries were prepared for Illumina sequencing according to Chatterjee et al. [31] and sequenced on a MiSeq System (Illumina) with custom primers. The sequence of linker oligonucleotides and primers used are given in S7 Table. To determine the genome-wide integration profiles raw sequence reads were processed through a custom suite of Perl scripts [30] modified to accommodate sequences of Tf1-*nat*AI and Illumina technology. Maps of integration relative to ORFs and Sap1 motifs were performed according to previous work [32]. Density plots were obtained using the R function densCol from the package grDevices [119]. R: A language and environment for statistical computing. R Foundation for Statistical Computing, Vienna, Austria. URL https://www.R-project.org/). The sequence data can be obtained from the SRA database with the accession SRA Study: SRP100942.

### Gene ontology enrichment of genes that promote late stages of retrotransposition in *S*. *cerevisiae* and *S*. *pombe*

The Biological Process slim terms optimized for *S*. *pombe* were applied to genes important for late stages of transposition of Tf1, Ty1 and Ty3. Term enrichments were calculated against the list of non-essential genes available from the Bioneer *S*. *pombe* deletion library and the *S*. *cerevisiae* ORF deletion collection in strain BY4741 from Invitrogen MapPairs. The p-values were calculated using hypergeometric distance and corrected for multiple comparison with false discovery rate.

### Two-hybrid assays

Full-length Tf1 IN was fused to the C-terminus of a truncated DNA binding domain of LexA by ligating the IN sequence into the EcoRI and SalI sites of pSH2-1 [120]. Full-length Tf1 IN was also fused to the C-terminus of the Gal4 activation domain by ligating IN sequence into the XhoI site of pACT [121]. The host strain used in the two-hybrid screen was *S*. *cerevisiae* strain CTY10-5d (*MATα ade2 trp1-901 leu2-3*,*112 his3-200 gal4 gal80 URA3*::*lexAop-lacZ ura3-52*) [122]. The two-hybrid assays detected production of lacZ by lifting colonies to 3MM nitrocellulose filter (Whatman) that was then stored at -80°C overnight. The filters were thawed and at room temperature tested for galactosidase activity using X-gal [122]. The sequences of Cwf3 (amino acids 3–284) and Rhp18 (amino acids 16–308) were inserted into pACT.

## Supporting information

S1 Fig The construction of Tf1-*nat*AI. Tf1 expressed from the *nmt1* promoter contained *neo* as a selection marker. AsiSI and NotI restriction sites were used to replace *neo* with a version of *nat* disrupted with an artificial intron (*nat*AI).
(PDF)

S2 Fig Key for scoring results of the transposition assay. Patches of deletion strains were scored on a scale of 0 to 5, with 5 being the growth of wild-type cells. Shown is a series of four deletion strains and the arrows indicate the score associated with specific patches that were used as standards for scoring all the deletion strains.
(PDF)

S3 Fig Key for scoring results of the homologous recombination assay that relied on cell patches. Patches of deletion strains were scored on a scale of 0 to 5, with 5 being the growth of wild-type cells. Shown is a series of four deletion strains and the arrows indicate the score associated with specific patches that were used as standards for scoring all the deletion strains.
(PDF)

S4 Fig A quantitative version of the homologous recombination assay can precisely measure reductions in activity. Tf1-*nat*AI is expressed in deletion strains by growing cells in liquid media lacking vitamin B1 (PM-U-B1+Leu). The cells are subsequently diluted and spread onto agar containing YES to count viable cells, and on agar containing YES+Nat to count cells with recombination.
(PDF)

S5 Fig Transposition assays of Tf1 with single amino acid substitutions in the catalytic core domain (CC). Four independent transformants of each mutation were tested. Panel A contains transposition patches for D987N and D1047N. Panel B contains transposition patches for E1083Q.
(PDF)

S6 Fig Immunoblots of Tf1 with single amino acid substitutions in the catalytic core domain (CC). A. Blot of independent transformants of mutants D1047N and D987N. B. Blot of independent transformants of E1083Q. Both blots were probed with polyclonal antibodies raised against IN and Gag.
(PDF)

S7 Fig Gene ontology enrichment of genes that promote late stages of retrotransposition in *S*. *cerevisiae* and *S*. *pombe*. The Biological Process slim terms (Accession # GO:0006260) of non-essential genes included in the deletion sets optimized for *S*. *pombe* were applied to genes important for late stages of transposition (S4 Table). The asterisk indicates p values <0.05 for hypergeometric distance and FDR correction. The color of the asterisk corresponds to the different retrotransposons. Tf1, blue, Ty1, red, and Ty3, green.
(PDF)

S8 Fig DNA blot measurements of Tf1 cDNA produced by strains with deletion mutations. DNA was extracted from wild-type and deletion strains expressing Tf1-*nat*AI. The DNA was digested with BsrGI and analyzed by DNA blot using a probe of *nat* sequence. The levels of Tf1 cDNA (2.9 kb) relative to plasmid (14 kb) were quantified by phosphoimaging.
(PDF)

S1 Table The transposition and recombination scores for each strain in the deletion collection provided in Excel. (XLSX)

S2 Table The deletion strains with defects in transposition and recombination provided in Excel. (XLSX)

S3 Table Quantitative recombination and immunoblot data for integration factor candidates provided in Excel. (XLSX)

S4 Table Gene ontology terms for biological function of factors that support transposition of Tf1, Ty1, and Ty3. (PDF)

S5 Table Summary statistics for integration sites of strains lacking *nup61*, *pht1*, *snf5*, *cwf12*, *rad51*, *rad50*, *or set1*. (PDF)

S6 Table Plasmids used in this study. (PDF)

S7 Table Oligonucleotides used in this study. (PDF)

S8 Table Yeast strains used in this study. (PDF)
