# Supplementary figures and images for: Host factors that promote retrotransposon integration are similar in distantly related eukaryotes
Source: PLoS Genet. 2017 Dec 12;13(12):e1006775. doi: 10.1371/journal.pgen.1006775 (PMC5741268; doi:10.1371/journal.pgen.1006775)

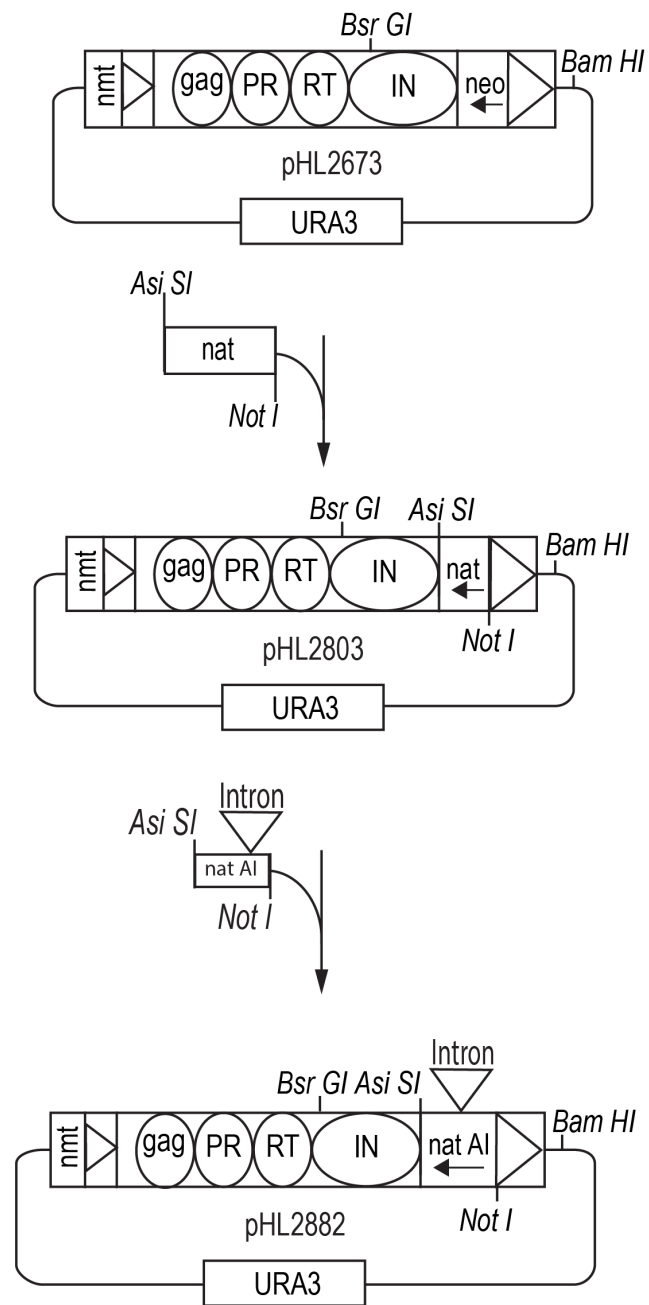

Fig.S1

Supplement: S1 Fig — Tf1 expressed from the nmt1 promoter contained neo as a selection marker. AsiSI and NotI restriction sites were used to replace neo with a version of nat disrupted with an artificial intron (natAI). (PDF) [file pgen.1006775.s001.pdf]

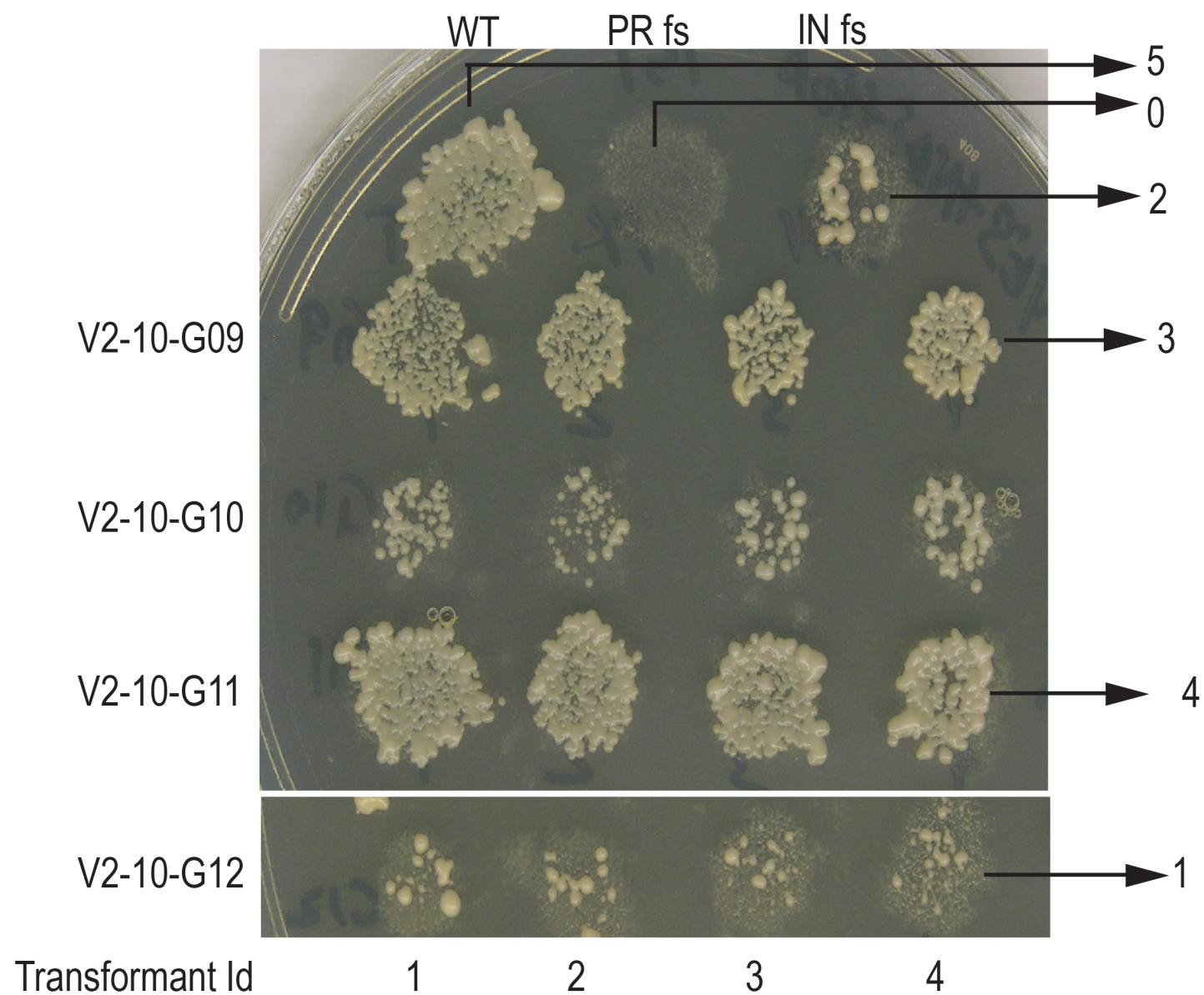

Fig.S2

Supplement: S2 Fig — Patches of deletion strains were scored on a scale of 0 to 5, with 5 being the growth of wild-type cells. Shown is a series of four deletion strains and the arrows indicate the score associated with specific patches that were used as standards for scoring all the deletion strains. (PDF) [file pgen.1006775.s002.pdf]

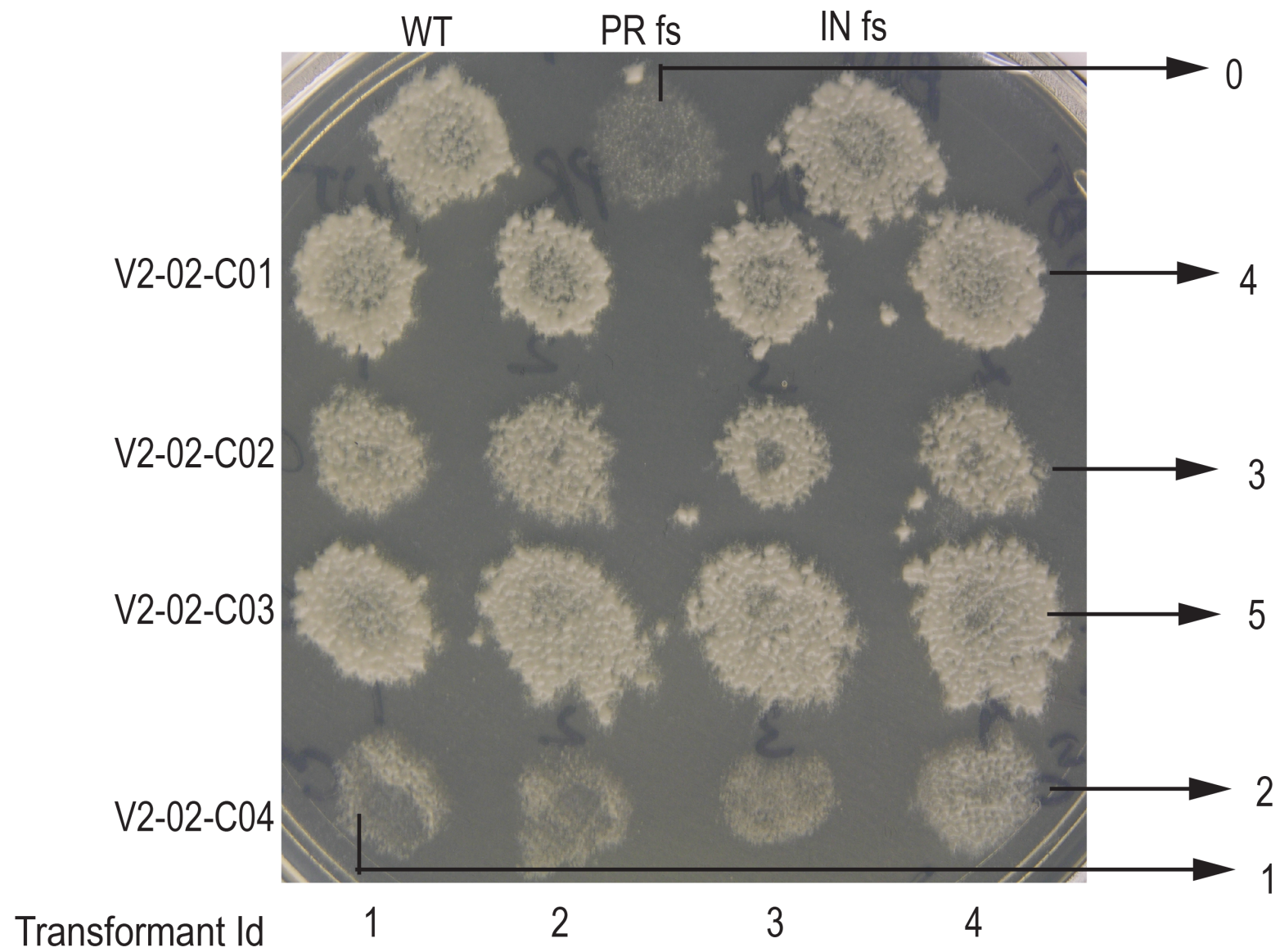

Fig.S3

Supplement: S3 Fig — Patches of deletion strains were scored on a scale of 0 to 5, with 5 being the growth of wild-type cells. Shown is a series of four deletion strains and the arrows indicate the score associated with specific patches that were used as standards for scoring all the deletion strains. (PDF) [file pgen.1006775.s003.pdf]

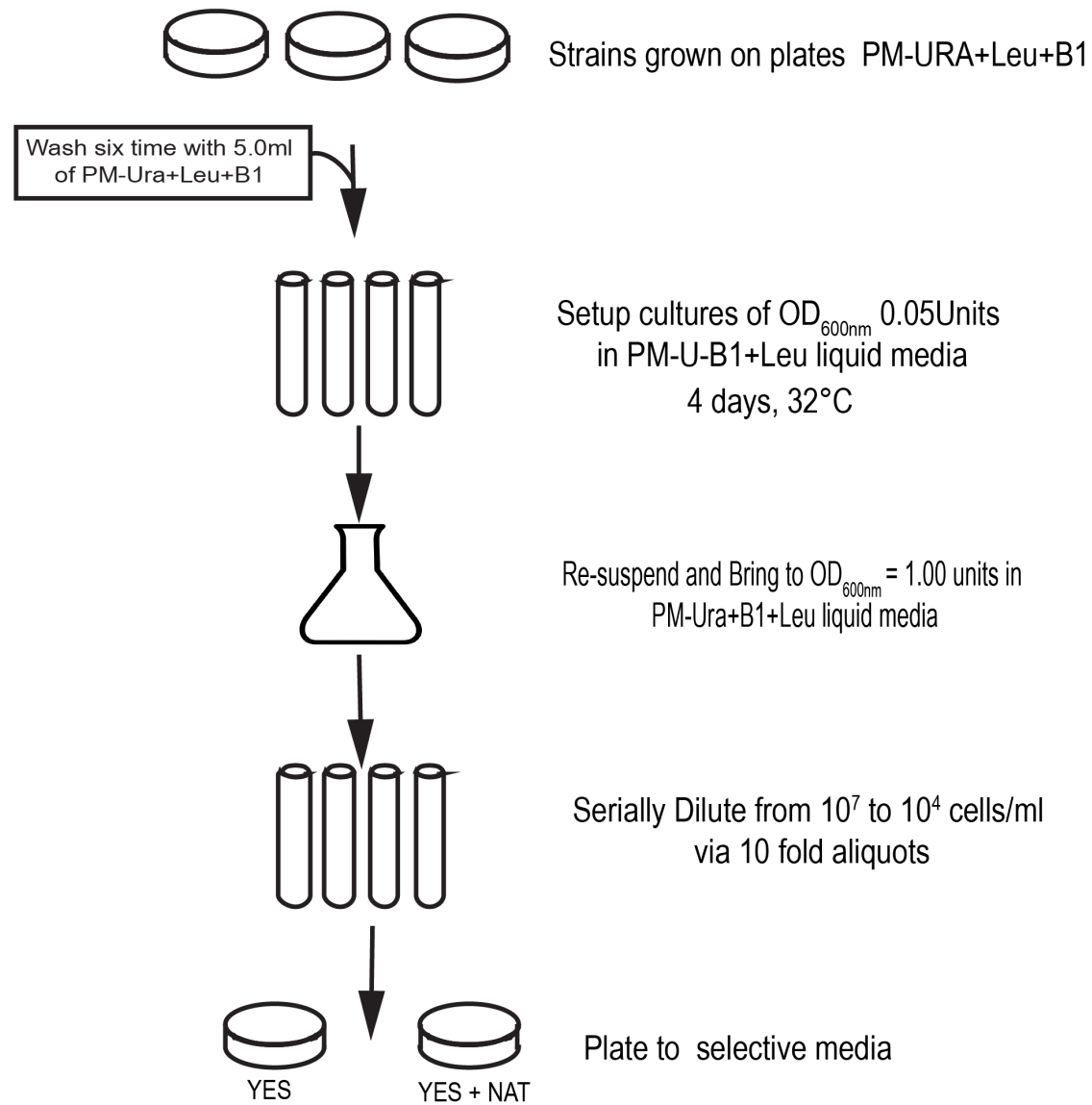

Fig.S4

Supplement: S4 Fig — Tf1-natAI is expressed in deletion strains by growing cells in liquid media lacking vitamin B1 (PM-U-B1+Leu). The cells are subsequently diluted and spread onto agar containing YES to count viable cells, and on agar containing YES+Nat to count cells with recombination. (PDF) [file pgen.1006775.s004.pdf]

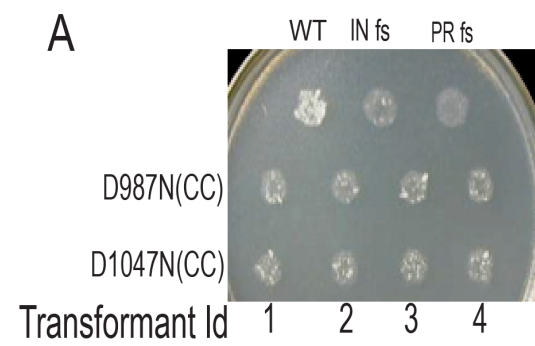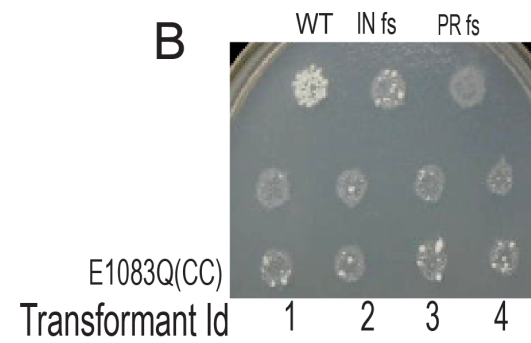

Fig.S5

Supplement: S5 Fig — Four independent transformants of each mutation were tested. Panel A contains transposition patches for D987N and D1047N. Panel B contains transposition patches for E1083Q. (PDF) [file pgen.1006775.s005.pdf]

A

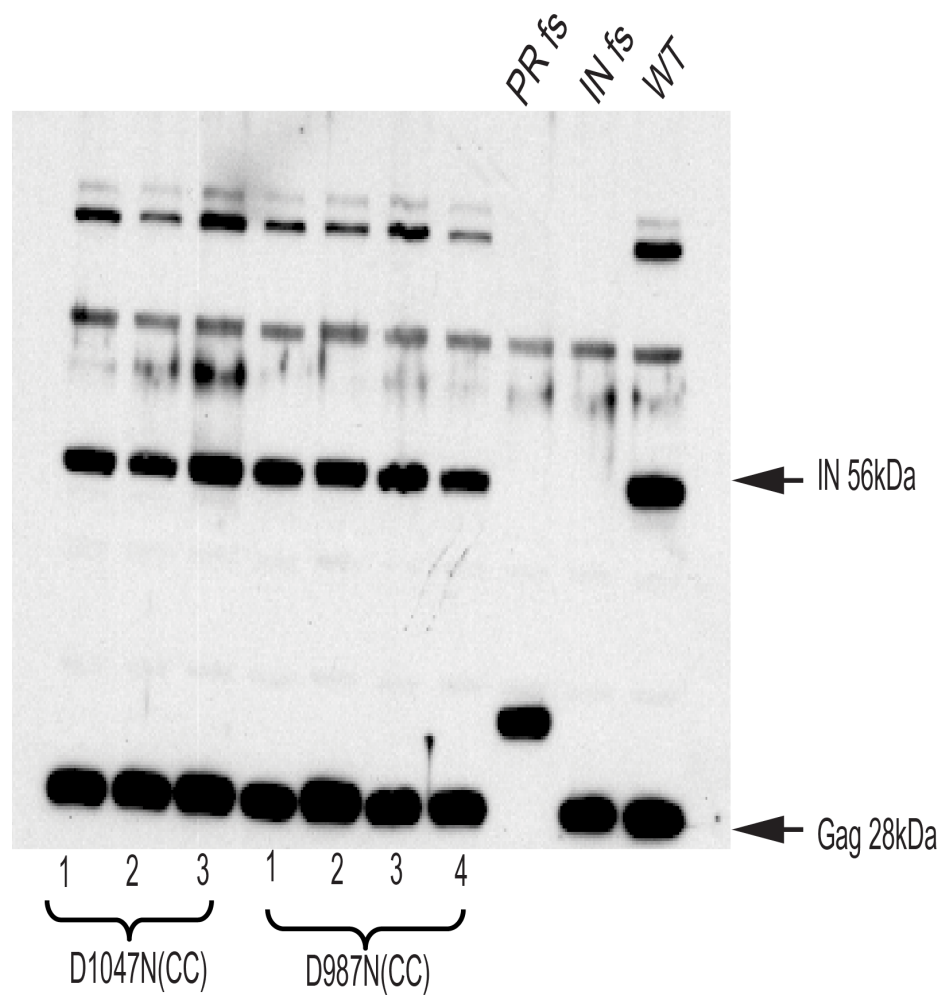

B

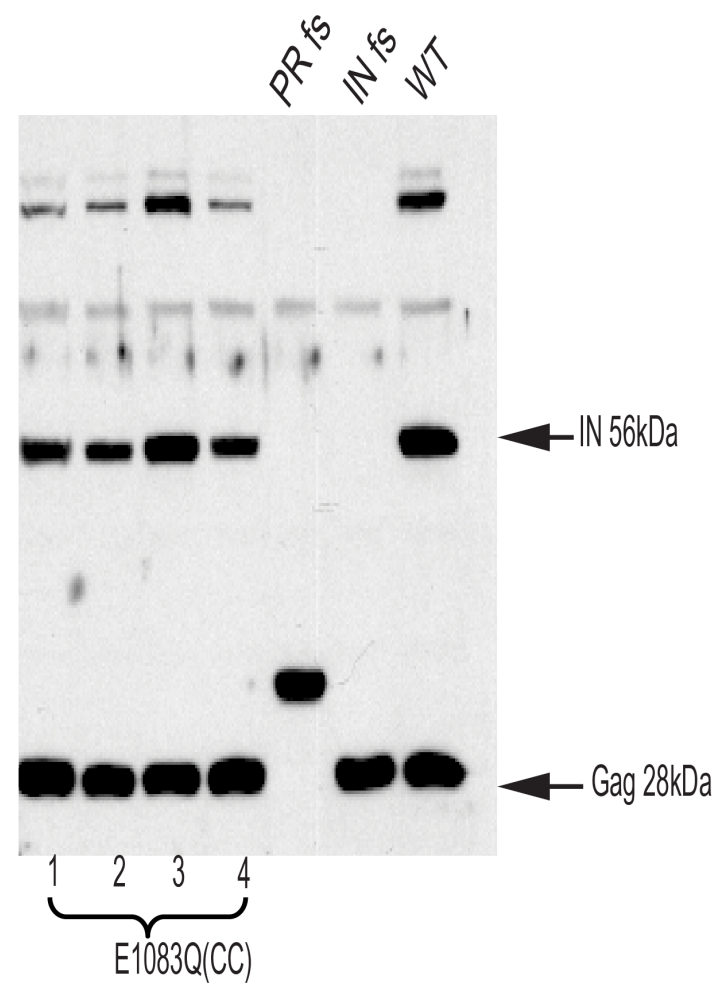

Fig.S6

Supplement: S6 Fig — A. Blot of independent transformants of mutants D1047N and D987N. B. Blot of independent transformants of E1083Q. Both blots were probed with polyclonal antibodies raised against IN and Gag. (PDF) [file pgen.1006775.s006.pdf]
