# Supplementary material for: Host factors that promote retrotransposon integration are similar in distantly related eukaryotes
Source: PLoS Genet. 2017 Dec 12;13(12):e1006775. doi: 10.1371/journal.pgen.1006775 (PMC5741268; doi:10.1371/journal.pgen.1006775)
Supplement: S7 Fig — The Biological Process slim terms (Accession # GO:0006260) of non-essential genes included in the deletion sets optimized for S. pombe were applied to genes important for late stages of transposition (S4 Table). The asterisk indicates p values <0.05 for hypergeometric distance and FDR correction. The color of the asterisk corresponds to the different retrotransposons. Tf1, blue, Ty1, red, and Ty3, green. (PDF) [file pgen.1006775.s007.pdf]

Fold-enrichment of factors that promote late stages of transposition for Tf1, Ty1 and Ty3 using GO slim terms of *S. pombe*

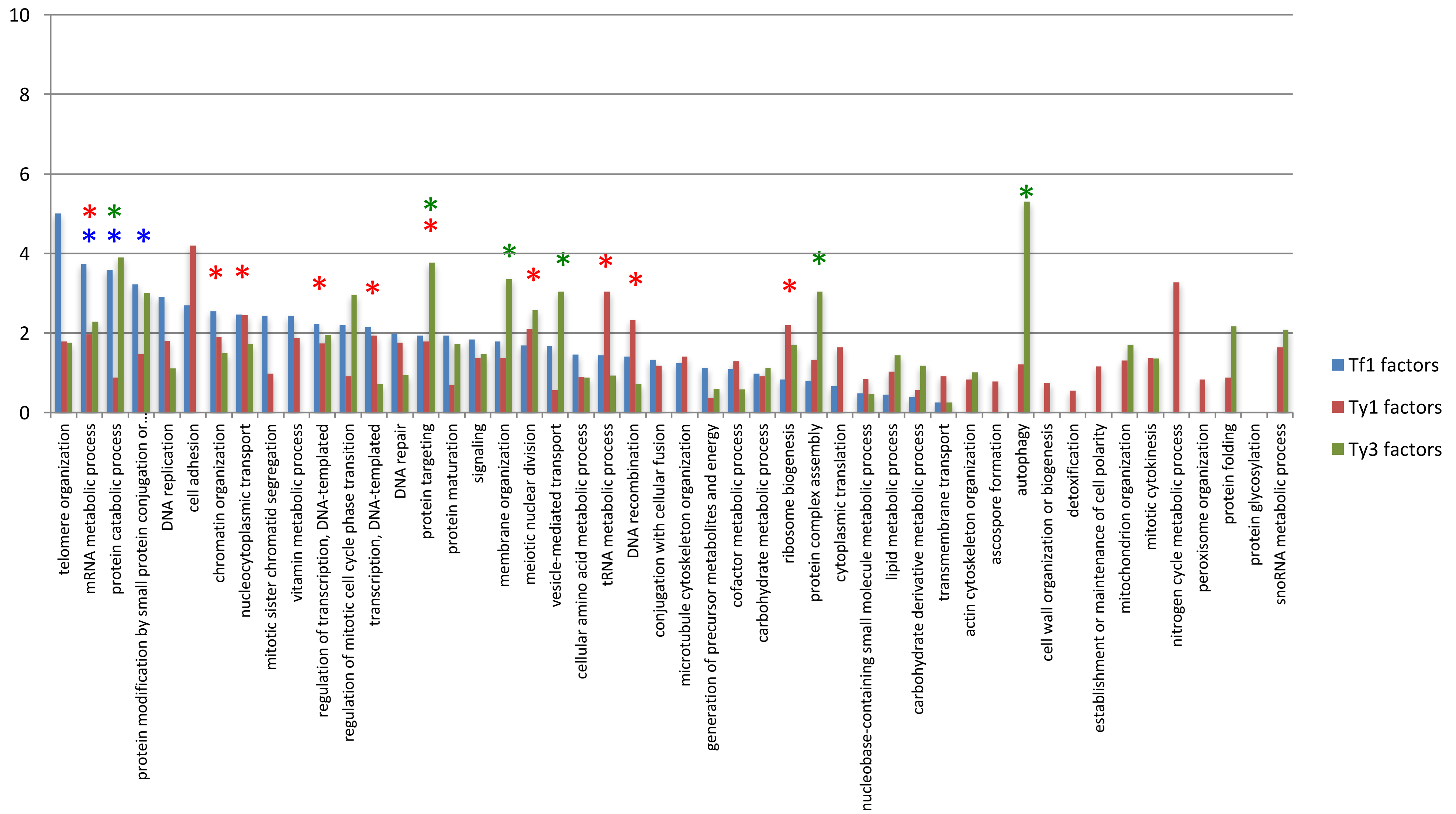

*p values <0.05 for Hypergeometric distance and FDR correction. The color corresponds to the different retrotransposons.

Suppl. Fig. S7
