# Supplementary material for: Host factors that promote retrotransposon integration are similar in distantly related eukaryotes
Source: PLoS Genet. 2017 Dec 12;13(12):e1006775. doi: 10.1371/journal.pgen.1006775 (PMC5741268; doi:10.1371/journal.pgen.1006775)
Supplement: S8 Fig — DNA was extracted from wild-type and deletion strains expressing Tf1-natAI. The DNA was digested with BsrGI and analyzed by DNA blot using a probe of nat sequence. The levels of Tf1 cDNA (2.9 kb) relative to plasmid (14 kb) were quantified by phosphoimaging. (PDF) [file pgen.1006775.s008.pdf]

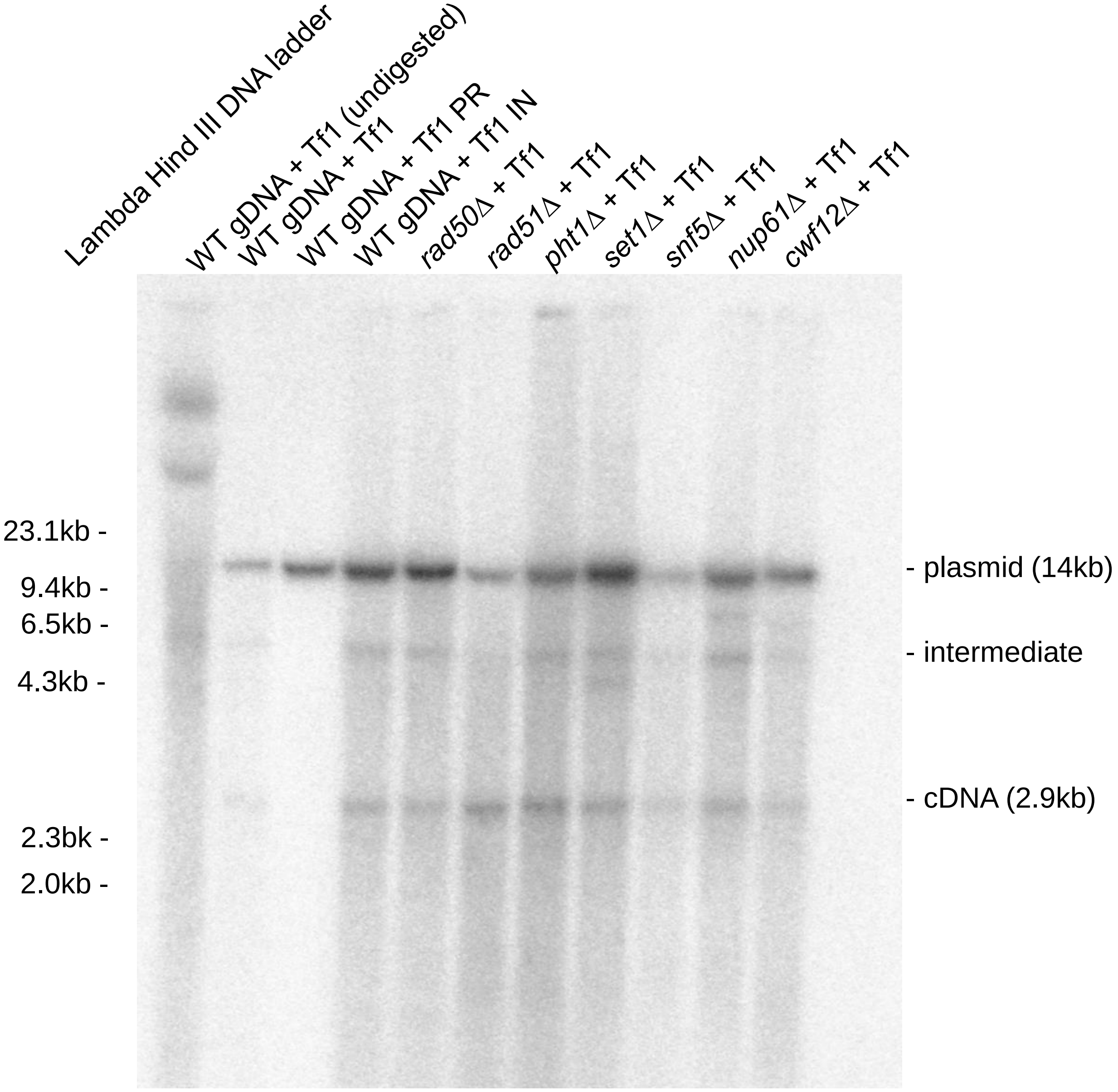

**Strain background**	WT	WT	WT	rad50Δ	rad51Δ	pht1Δ	set1Δ	snf5Δ	nup61Δ	cwf12Δ
**Tf1**	+	PRfs	INfs	+	+	+	+	+	+	+
**Relative level of cDNA**	0.23	0.01	0.24	0.22	0.73	0.40	0.25	0.40	0.37	0.28

Supplementary Figure S8
