# Supplementary material for: Host factors that promote retrotransposon integration are similar in distantly related eukaryotes
Source: PLoS Genet. 2017 Dec 12;13(12):e1006775. doi: 10.1371/journal.pgen.1006775 (PMC5741268; doi:10.1371/journal.pgen.1006775)
Supplement: S4 Table — (PDF) [file pgen.1006775.s012.pdf]

Suppl. Table S4: Fission Yeast gene ontology slim terms

**Biological function**	**Tf1 factors**	**Ty1 factors [1, 2, 3]**	**Ty3 factors [4, 5]**
regulation of transcription, DNA-templated (GO:0006355)	SPAC13A11.04c, SPAC1851.03, SPAC20H4.03c, SPAC2F7.08c, SPAC31G5.12c, SPAPB17E12.04c, SPBC12D12.06, SPBC19C2.14, SPBC947.10, SPCC306.04c, SPCC757.04	YAL013W, YAL021C, YBL079W, YBR215W, YBR245C, YCR081W, YDL116W, YDR123C, YDR207C, YDR334W, YDR457W, YDR485C, YER028C, YFL031W, YFL044C, YGR200C, YGR229C, YHR167W, YJL089W, YJL127C, YJL179W, YKL020C, YKL057C, YKL110C, YKL160W, YKR082W, YLR039C, YLR052W, YLR182W, YLR357W, YLR384C, YLR445W, YMR039C, YMR069W, YMR312W, YNL021W, YNL097C, YNL288W, YNR052C, YOL004W, YOL067C, YOL108C, YPL055C, YPL086C, YPL101W, YPL139C, YPL230W, YPR179C	YAL013W, YAR003W, YBR103W, YDL088C, YGL173C, YIL035C, YJR102C, YLR417W, YML121W, YNL236W, YPL002C
chromatin organization (GO:0006325)	SPAC13A11.04c, SPAC2F7.08c, SPAC664.03, SPAPB17E12.04c, SPBC11B10.10c, SPBC19C2.14, SPBC36B7.08c, SPCC24B10.19c, SPCC306.04c	YAL013W, YBL079W, YBR215W, YBR245C, YDL116W, YDR207C, YDR334W, YDR485C, YJL127C, YKL020C, YKL057C, YKL160W, YKR082W, YLR052W, YLR319C, YLR357W, YMR069W, YNL021W, YNL097C, YOL004W, YPL055C, YPL086C, YPL101W, YPL139C, YPR179C	YAL013W, YAR003W, YBR103W, YML121W
protein catabolic process (GO:0030163)	SPAC1142.07c, SPAC13A11.04c, SPBC1734.06, SPBC215.14c, SPBC2D10.12, SPBC947.10, SPCC188.08c, SPCC338.16	YBR114W, YDR457W, YER098W, YFL016C, YFL044C, YHR200W, YIR025W, YKL010C, YNR075W, YPL084W	YER151C, YIL097W, YJR102C, YLR025W, YLR417W, YMR077C, YPL002C, YPL065W, YPL084W
protein modification by small protein conjugation or removal (GO:0070647)	SPAC13A11.04c, SPAC16A10.03c, SPAPB17E12.04c, SPBC1734.06, SPBC2D10.12, SPBC947.10, SPCC188.08c, SPCC338.16	YBL069W, YBL079W, YDL115C, YDL116W, YDL142C, YGR133W, YKL057C, YKR082W, YLR011W, YML103C, YML105C, YMR060C, YNL121C, YNL329C, YNR075W, YOL111C, YOR045W, YPL084W, YPL195W, YPR049C, YPR133W-	YDR139C, YER151C, YIL073C, YIL097W, YPL084W

		A	
mRNA metabolic process (GO:0016071)	SPAC1D4.11c, SPAC20H4.03c, SPBC19C2.14, SPBC19G7.10c, SPBC32F12.05c, SPCC1919.05, SPCC825.05c	YAL021C, YDL160C, YDR378C, YGL213C, YJL124C, YJL140W, YKL074C, YKL204W, YNL016W, YNL224C, YNL288W, YNL299W, YNR024W, YNR052C, YOR243C, YPL213W, YPR101W	YDL160C, YFL001W, YGL173C, YNL224C
signaling (GO:0023052)	SPAC1851.03, SPAC30.01c, SPBC1539.08, SPBC947.10, SPBP23A10.10, SPCC162.12, SPCC285.09c	YBR077C, YDL006W, YDR477W, YER027C, YFL031W, YFL044C, YFR008W, YGR014W, YGR092W, YGR133W, YHR082C, YIL047C, YKL178C, YLR187W, YLR425W, YNL127W, YNL329C, YOL067C, YOL105C, YOR267C, YOR360C, YPL268W, YPR075C	YBR103W, YHR082C, YML121W, YMR116C, YPL150W
vesicle-mediated transport (GO:0016192)	SPAC1142.07c, SPAC16A10.03c, SPAC30.01c, SPAC31A2.13c, SPBC1539.08, SPBC215.14c	YDR320C, YER122C, YGL054C, YKL129C, YLR039C, YNL079C, YNR075W, YPL084W, YPL195W	YBL007C, YER151C, YFR019W, YGL212W, YGR167W, YLR025W, YLR242C, YMR077C, YPL084W, YPR173C
DNA repair (GO:0006281)	SPAC1556.01c, SPAC644.14c, SPBC1734.06, SPBC2D10.12	YBR114W, YDL116W, YDR369C, YER041W, YHL022C, YJL127C, YJL140W, YJR043C, YKL057C, YKR082W, YLR320W, YLR357W, YML032C, YMR039C, YNL082W, YNL085W, YOL004W, YOR368W	YAL027W, YER173W
cellular amino acid metabolic process (GO:0006520)	SPAC30C2.04, SPAC343.10, SPBC21C3.08c	YAL044C, YDR268W, YGL125W, YJR025C, YJR137C, YJR139C, YLR438W, YMR169C, YMR289W, YOL058W	YBR263W, YJR137C
DNA replication (GO:0006260)	SPAC1556.01c, SPAC644.14c, SPCC338.16	YAL013W, YAL021C, YCL016C, YFL036W, YJR043C, YLR320W, YOL004W, YPL055C	YAL013W
meiotic nuclear division (GO:0007126)	SPAC1556.01c, SPAC18G6.10, SPAC644.14c	YDR014W, YDR207C, YDR369C, YGL213C, YGL250W, YHL022C, YIR025W, YJR021C, YLR182W, YLR320W, YLR445W, YML032C, YNL082W, YOR368W, YPL139C, YPL157W	YBR103W, YER173W, YFR012W, YIL073C

membrane organization (GO:0061024)	SPAC1142.07c, SPAC18G6.10, SPBC215.14c	YBL069W, YBL079W, YDL142C, YDR375C, YER122C, YIL040W, YKL053C-A, YMR060C, YNL121C, YOL111C, YOR045W, YPL084W, YPR075C, YPR133W-A	YBR262C, YGL212W, YLR025W, YLR242C, YMR077C, YPL084W, YPR173C
mitotic sister chromatid segregation (GO:0000070)	SPAC6C3.08, SPBC2G2.14, SPBC646.13	YCL016C, YDR014W, YIR025W, YJL030W	
nucleocytoplasmic transport (GO:0006913)	SPBC1703.03c, SPCC1753.05, SPCC18B5.07c	YBL079W, YDL115C, YDL116W, YDR457W, YGR285C, YHR167W, YIL040W, YJL140W, YKL057C, YKR082W, YLR011W, YML103C, YNR034W, YOL121C	YDL088C, YPL125W
protein targeting (GO:0006605)	SPAC1142.07c, SPBC1703.03c, SPBC215.14c	YAL021C, YIR025W, YJL030W	YDL088C, YGL212W, YJR102C, YLR417W, YOL083W, YPL002C, YPL065W, YPL084W, YPL125W
regulation of mitotic cell cycle phase transition (GO:1901990)	SPAC1D4.11c, SPAC6C3.08, SPBC646.13		YER173W, YGL173C
telomere organization (GO:0032200)	SPAC1556.01c, SPAC644.14c, SPCC338.16	YDR369C, YHR167W, YLR052W, YML032C, YPL157W	YAR003W
carbohydrate metabolic process (GO:0005975)	SPAC19G12.15c, SPAC3G6.09c	YAL013W, YDR207C, YDR477W, YHR104W, YIL045W, YIL155C, YJL089W, YKR097W, YNR034W, YNR067C, YOL030W, YPR049C	YAL013W, YGL156W, YIL097W
cofactor metabolic process (GO:0051186)	SPAC1805.06c, SPAC343.10	YGL125W, YIL155C, YJR025C, YJR122W, YKR072C, YLR239C, YMR169C, YMR289W, YNR034W, YOL055C, YPL135W	YBR263W
conjugation with cellular fusion (GO:0000747)	SPBC646.13, SPCC285.09c	YDL006W, YFR008W, YIL037C, YKL178C, YNL127W, YOL111C, YPR075C	
cytoplasmic	SPAC30C2.04, SPCC24B10.09	YDL081C, YDR025W, YDR152W, YER0	

translation (GO:0002181)		56C-A, YHR021C, YHR168W, YKL006W, YLR185W, YNL069C, YOL121C, YOR234C, YPL079W	
DNA recombination (GO:0006310)	SPAC1556.01c, SPAC644.14c	YDR369C, YFR038W, YGL213C, YGL250W, YHL022C, YHR167W, YIR025W, YJR021C, YJR043C, YLR052W, YLR182W, YLR320W, YLR357W, YLR445W, YML032C, YOR368W	YER173W
ribosome biogenesis (GO:0042254)	SPBC1703.03c, SPCC24B10.09	YDR025W, YDR237W, YDR378C, YDR457W, YER056C-A, YGL078C, YGR285C, YHR021C, YKL006W, YKL057C, YKR060W, YLR185W, YLR221C, YNL224C, YNR024W, YOL041C, YOL121C, YOR234C, YOR243C	YGL078C, YGL173C, YNL224C
transcription, DNA-templated (GO:0006351)	SPAC20H4.03c, SPAC664.03	YAL013W, YAL021C, YBR215W, YBR245C, YCR081W, YDR123C, YDR207C, YDR334W, YDR457W, YER028C, YFL031W, YFL036W, YGR200C, YGR229C, YHR167W, YJL089W, YJL140W, YKL110C, YKL160W, YLR039C, YLR052W, YLR182W, YLR357W, YLR384C, YLR445W, YMR039C, YMR312W, YNL021W, YNL097C, YNL288W, YNR052C, YOL004W, YOL067C, YOL108C, YPL055C, YPL086C, YPL101W, YPL139C, YPL230W, YPR179C	YAL013W, YML121W, YNL236W
tRNA metabolic process (GO:0006399)	SPAC30C2.04, SPBC1861.05	YDL006W, YDL033C, YDR268W, YDR378C, YGR200C, YJL098W, YKL110C, YLR384C, YMR283C, YMR312W, YNL299W, YOR243C, YPL086C, YPL101W, YPL135W, YPL157W	YFL001W
carbohydrate derivative metabolic process (GO:1901135)	SPCC285.09c	YBL099W, YIL155C, YJR105W, YKR072C, YML106W, YNR034W, YOR270C	YGL156W, YLR242C, YOR270C

cell adhesion (GO:0007155)	SPAC2F7.08c	YDR477W, YER027C, YIR019C	
generation of precursor metabolites and energy (GO:0006091)	SPCC794.12c	YIL045W, YKL055C, YPR049C	YJR120W
lipid metabolic process (GO:0006629)	SPCC594.04c	YAL013W, YCL026C-A, YDL142C, YDR123C, YDR207C, YGR157W, YHR133C, YJL196C, YKL020C, YKL055C, YLL012W, YLR239C, YOL108C, YPL268W	YAL013W, YFR019W, YLR242C, YPR173C
microtubule cytoskeleton organization (GO:0000226)	SPBC2G2.14	YJR070C, YLR319C, YPL152W, YPL174C	
nucleobase-containing small molecule metabolic process (GO:0055086)	SPCC285.09c	YBL099W, YEL016C, YIL155C, YJR025C, YJR105W, YKR072C, YML106W, YNR034W, YOR270C	YOR270C
protein complex assembly (GO:0006461)	SPAC31G5.12c	YBL079W, YBR245C, YDR375C, YDR435C, YER027C, YGL213C, YHR200W, YKL010C, YKL053C-A, YML105C, YMR039C, YMR060C, YOR045W, YOR270C, YPR133W-A	YBL007C, YDR493W, YGL173C, YGR167W, YOL083W, YOR270C, YPR173C
protein maturation (GO:0051604)	SPBC947.10	YBR114W, YDR457W, YER098W, YFL044C, YGR133W, YGR200C, YIR025W, YKL010C, YLR445W, YMR312W, YPL055C, YPL084W	YMR154C
transmembrane transport (GO:0055085)	SPBC725.10	YBL099W, YBR068C, YDR270W, YDR375C, YER060W, YGR133W, YHL016C, YHR026W, YJL129C, YML105C, YMR060C, YNL121C, YNL268W, YNL329C, YOR045W, YOR270C, YPR003C, YPR133W-A	YOR270C
vitamin metabolic process (GO:0006766	SPBC26H8.01	YMR169C, YMR289W, YOL055C, YPR121W	

)			
actin cytoskeleton organization (GO:0030036)		YFR024C-A, YLR319C, YNL079C, YOR304C-A	YBL007C
ascospore formation (GO:0030437)		YGL054C, YJL038C, YMR039C, YOR242C	
autophagy (GO:0006914)		YBR077C, YDR435C, YDR477W, YHR082C, YKL006W, YNL127W, YOL105C, YPL166W, YPR049C	YER151C, YGL212W, YHR082C, YLR417W, YLR423C, YML121W, YOL083W, YPR173C
cell wall organization or biogenesis (GO:0071554)		YBR301W, YDR477W, YGR014W, YGR229C, YJL038C, YKL129C, YLR425W, YOL030W, YOL105C, YOR242C	
detoxification (GO:0098754)		YKR066C	
establishment or maintenance of cell polarity (GO:0007163)		YGL054C, YGR014W, YKL129C, YLR319C, YNL079C, YPL174C	
mitochondrion organization (GO:0007005)		YDL006W, YDL033C, YDL142C, YDR375C, YFL016C, YFL036W, YJL063C, YKL053C-A, YMR060C, YNL079C, YNL121C, YOR045W, YPL166W, YPR049C, YPR133W-A	YBR262C, YDR493W, YJR120W, YLR423C
mitotic cytokinesis (GO:0000281)		YDL117W, YGL054C, YGR014W, YKL129C, YLR319C	YFR012W
nitrogen cycle metabolic process (GO:0071941)		YHL016C, YOL058W	
peroxisome organization (GO:0007031)		YGR133W, YNL329C	
protein folding (GO:0006457)		YFL016C, YGR285C, YJL179W, YJR032W	YDR493W, YJR032W

protein glycosylation (GO:0006486)		YBR106W, YPL135W	
snoRNA metabolic process (GO:0016074)		YNL299W	YGL173C

[1] (GRIFFITH et al. 2003)
[2] (DAKSHINAMURTHY et al. 2010)
[3] (RISLER et al. 2012)
[4] (IRWIN et al. 2005)
[5] (AYE et al. 2004)
