# Supplementary material for: Host factors that promote retrotransposon integration are similar in distantly related eukaryotes
Source: PLoS Genet. 2017 Dec 12;13(12):e1006775. doi: 10.1371/journal.pgen.1006775 (PMC5741268; doi:10.1371/journal.pgen.1006775)
Supplement: S5 Table — (PDF) [file pgen.1006775.s013.pdf]

| Locations | WT | WT2 | ***nup61*Δ** | ***pht1*Δ** | ***snf5*Δ** | ***cwf12*Δ** | ***rad51*Δ** | ***rad50*Δ** | ***set1*Δ** |
|---|---|---|---|---|---|---|---|---|---|
| | | Number of total insertions | | | | | | | |
| | 49,240 | 45,536 | 41,459 | 15,304 | 39,856 | 24,478 | 13,915 | 47,705 | 41,605 |
| | | % of total insertions | | | | | | | |
| upstream | 78.81 | 79.60 | 75.14 | 79.69 | 78.71 | 78.85 | 80.34 | 78.75 | 78.37 |
| downstream | 16.56 | 16.46 | 16.38 | 16.09 | 16.93 | 16.48 | 15.54 | 16.87 | 17.61 |
| in ORF | 4.63 | 3.95 | 8.47 | 4.21 | 4.35 | 4.67 | 4.13 | 4.38 | 4.01 |
| tandem | 41.64 | 42.14 | 40.11 | 40.24 | 42.22 | 41.09 | 40.90 | 41.81 | 42.07 |
| divergent | 49.87 | 50.24 | 47.35 | 51.69 | 49.46 | 50.34 | 51.57 | 49.82 | 49.58 |
| convergent | 3.41 | 3.50 | 3.66 | 3.59 | 3.61 | 3.35 | 3.30 | 3.61 | 3.94 |

**Supplementary Table S5. Positions of integration sites.**
