# Supplementary material for: Host factors that promote retrotransposon integration are similar in distantly related eukaryotes
Source: PLoS Genet. 2017 Dec 12;13(12):e1006775. doi: 10.1371/journal.pgen.1006775 (PMC5741268; doi:10.1371/journal.pgen.1006775)
Supplement: S6 Table — (PDF) [file pgen.1006775.s014.pdf]

Suppl. Table S6: Plasmids

**Plasmid ID number**	**Plasmid Description**	**Purpose**	**Sources**
pHL415-2	Tf1-*neo* with PRfs	Construction of pHL2804	(LEVIN *et al.* 1993)
pHL431-25	Tf1-*neo* with INfs	Construction of pHL2805	(LIN AND LEVIN 1997)
pHL2597	nat fragment	Production of nat probe for Southern blot	(SATO *et al.* 2005)
pHL2673	Tf1-*neo* with U5 tag for deep sequencing inserts	Used for construction of plasmid with Tf1 marked with *nat*.	(GUO AND LEVIN 2010)
pHL2803	Tf1-*nat*	For construction of pHL2882	This study
pHL2804	Tf1-*nat* PRfs	For construction of pHL2883	This study
pHL2805	Tf1-*nat* INfs	For construction of pHL2884	This study
pHL2882	Tf1-*nat*AI	Expression of Tf1 transposon with *Nat*AI used to screen deletion strains	This study
pHL2883	Tf1-*nat*AI PRfs	Version of pHL2882 with PRfs	This study
pHL2884	Tf1-*nat*AI INfs	Version of pHL2882 with INfs	This study
pHL2898	Tf1-*nat*AI with D987N	Used to assay activity of D987N mutation in catalytic core of Tf1-IN	This study
pHL2900	Tf1-*neo*AI with D1047N	Used to assay activity of D1047N mutation in catalytic core of Tf1-IN	This study
pHL2902	Tf1-*neo*AI with E1083Q	Used to assay activity of E1083Q mutation in catalytic core of Tf1-IN	This study
