# Supplementary material for: Host factors that promote retrotransposon integration are similar in distantly related eukaryotes
Source: PLoS Genet. 2017 Dec 12;13(12):e1006775. doi: 10.1371/journal.pgen.1006775 (PMC5741268; doi:10.1371/journal.pgen.1006775)
Supplement: S7 Table — (PDF) [file pgen.1006775.s015.pdf]

Suppl. Table S7: Oligonucleotides

Oligo's ID	Oligonucleotide sequence	Oligo description
HL1319	GAAGGTAAATTGATACATCCAGGC	+strand primer upstream of the BsrGI site for PCR of D987N/D1047N/E1083Q mutations
HL1320	CGATGAGTTTTTCTAATCAGAATTGG	-strand primer downstream of the NarI site for PCR of D987N/D1047N/E1083Q mutations
HL1427	CCTTGGGAATCTTTATCAATGAATTTTATTACAGCCTTACC	Used in PCR with HL1320 for half fusion product with D987N mutation
HL1428	GGTAAGGCTGTAATAAAATTCATTGATAAAGATTCCCAAGG	Used in PCR with HL1319 for half fusion product with D987N mutation
HL1429	GCAATCCAAAAGAAATCATTGCAAATAATGATCATATTTTTACTTC	Used in PCR with HL1320 for half fusion product with D1047 N mutation
HL1430	GAAGTAAAAATATGATCATTATTTGCAATGATTTCTTTTGGATTGC	Used in PCR with HL1319 for half fusion product with D1047 N mutation
HL1431	CCACAAACTGATGGACAAACTCAACGTACAAACCAAACTGTGG	Used in PCR with HL1320 for half fusion product with E1083Q mutation
HL1432	CCACAGTTTGGTTTGTACGTTGAGTTTGTCCATCAGTTTGTGG	Used in PCR with HL1319 for half fusion product with E1083Q mutation
HL2627	CAAATACAAGAATATGTACAGAATTGCC	Upstream of BsrGI for construction of pHL2803
HL2628	GTGCTGCTAGCCAGCTGGGATCC	Downstream of BamHI for construction of pHL2803
HL2629	GCTCACCGCGGACTGCGGCCGCATCTTGGGGAGGGCAATG	+strand fusion primer to make AsiSI-SacII-NotI sites for pHL2803
HL2630	GCAGTCCGCGGTGAGCGATCGCTTTCTATGTAAATCTGAG	-strand fusion primer to make AsiSI-SacII-NotI sites for pHL2803
HL3484	GGACTACATTAGCTACAGCGCTACAAGC	Validation of Nat-AI insert in new version of Tf1 plasmid pHL2882

HL3485	CCGGCTCCCCTTTCCCGAG	Validation of Nat-AI insert in new version of Tf1 plasmid pHL2882
HL3486	CGCTGGTACGATATCTGTATGCAGTGTC	Validation of Nat-AI insert in new version of Tf1 plasmid pHL2882
HL3487	CGGGTATCTATGCATATAGAGCGG	Validation of Nat-AI insert in new version of Tf1 plasmid pHL2882
HL3488	CGCGTTGAATTGTCCCCACG	Validation of Nat-AI insert in new version of Tf1 plasmid pHL2882
HL3489	CCACCGAGTTCGCTCGGGAAAGGG	Validation of Nat-AI insert in new version of Tf1 plasmid pHL2882
HL1870	GTAATACGACTCACTATAGGGCTCCGCTTAAGGGAC	Linker for Illumina sequencing
HL1871	P- TAGTCCCTTAAGCGGAG-3AmMO	Linker for Illumina sequencing
HL2216	CAAGCAGAAGACGGCATACGAGCTCTTCCGATCTGTAATACGACTC ACTATAGGGC	Linker primer for Illumina sequecing
HL5043	AATGATACGGCGACCACCGAGATCTACACTCTTTCCCTACACGACGC TCTTCCGATCTACGTCTCACCGCAGTTGATGCATAGGAATTTAG	Tf1 primer with barcode “ACGT” for Illumina sequecing
HL5038	AATGATACGGCGACCACCGAGATCTACACTCTTTCCCTACACGACGC TCTTCCGATCTTGCACTCACCGCAGTTGATGCATAGGAATTTAG	Tf1 primer with barcode “TGCA” for Illumina sequecing
HL5039	AATGATACGGCGACCACCGAGATCTACACTCTTTCCCTACACGACGC TCTTCCGATCTGTACCTCACCGCAGTTGATGCATAGGAATTTAG	Tf1 primer with barcode “GTAC” for Illumina sequecing
HL5040	AATGATACGGCGACCACCGAGATCTACACTCTTTCCCTACACGACGC TCTTCCGATCTCATGCTCACCGCAGTTGATGCATAGGAATTTAG	Tf1 primer with barcode “CATG” for Illumina sequecing
