# Supplementary material for: Host factors that promote retrotransposon integration are similar in distantly related eukaryotes
Source: PLoS Genet. 2017 Dec 12;13(12):e1006775. doi: 10.1371/journal.pgen.1006775 (PMC5741268; doi:10.1371/journal.pgen.1006775)
Supplement: S8 Table — (PDF) [file pgen.1006775.s016.pdf]

Suppl. Table S8: Yeast strains

**Strain ID**	**Genotype**	**Plasmids**	**Plasmid Description**	**Purpose**	**Sources**
YHL151	ED668 h+, ade6-M216 ura4-D18 leu 1-32			Bioneer Wild type strain *h+*	(Kim *et al.* 2010)
YHL181	ED665 h-, ade6-M210 ura4-D18 leu 1-32			Bioneer Wild type strain *h-*	This study
YHL 10287	ED668 h+, ade6-M216 ura4-D18 leu 1-32	pHL2882	Wild type Tf1-*nat AI*	Control strain TA and HRA assay	This study
YHL 10311	ED668 h+, ade6-M216 ura4-D18 leu 1-32	pHL2883	Tf1-*natAI* with PR frameshift	Control strain TA and HRA assay	This study
YHL10335	ED668 h+, ade6-M216 ura4-D18 leu 1-32	pHL2884	Tf1-*natAI* with IN frameshift	Control strain TA and HRA assay	This study
YHL10708	h+, ura4-D18, leu1-32, ade6-m210	pHL2898	Mutation in catalytic domain of Tf1-IN domain pHL2898	D987N mutation in catalytic core of Tf1-IN	This study
YHL10709	h+, ura4-D18, leu1-32, ade6-m210	pHL2898	Mutation in catalytic domain of Tf1-IN domain pHL2898	D987N mutation in catalytic core of Tf1-IN	This study
YHL10712	h+, ura4-D18, leu1-32, ade6-m210	pHL2900	Mutation in catalytic domain of Tf1-IN domain pHL2900	D1047 N mutation in catalytic core of Tf1-IN	This study
YHL10713	h+, ura4-D18, leu1-32, ade6-m210	pHL2900	Mutation in catalytic domain of Tf1-IN domain pHL2900	D1047 N mutation in catalytic core of Tf1-IN	This study
YHL10716	h+, ura4-D18, leu1-32, ade6-m210	pHL2902	Mutation in catalytic domain of Tf1-IN domain pHL2902	E1083Q mutation in Catalytic Core of Tf1-IN	This study

YHL10717	h+, ura4-D18, leu1-32, ade6-m210	pHL2902	Mutation in catalytic domain of Tf1-IN domain pHL2902	E1083Q mutation in Catalytic Core of Tf1-IN	This study
YHL12061	*h+ ade6-M216 ura4-D18 leu1-32*	pHL2882	Wild-type Tf1-natAI	Sequencing of integration profile in wild type and control strain Southern blot	This study
YHL12062	*h+ ade6-M216 ura4-D18 leu1-32*	pHL2883	Tf1-natAI with PR frameshift	Control strain Southern blot	This study
YHL12063	*h+ ade6-M216 ura4-D18 leu1-32*	pHL2884	Tf1-natAI with IN frameshift	Control strain Southern blot	This study
YHL12064	*h+ ade6-M216 ura4-D18 leu1-32 nup61Δ*	pHL2882	Wild-type Tf1-natAI	Sequencing of integration profile in nup61Δ strain	This study
YHL12065	*h+ ade6-M216 ura4-D18 leu1-32 snf5Δ*	pHL2882	Wild-type Tf1-natAI	Sequencing of integration profile in snf5Δ strain	This study
YHL12066	*h+ ade6-M216 ura4-D18 leu1-32 rad50Δ*	pHL2882	Wild-type Tf1-natAI	Sequencing of integration profile in rad50Δ strain	This study
YHL12067	*h+ ade6-M216 ura4-D18 leu1-32 set1Δ*	pHL2882	Wild-type Tf1-natAI	Sequencing of integration profile in set1Δ strain	This study
YHL12068	*h+ ade6-M210 ura4-D18 leu1-32 pht1Δ*	pHL2882	Wild-type Tf1-natAI	Sequencing of integration profile in pht1Δ strain	This study

YHL12069	*h+ ade6-M216 ura4-D18 leu1-32 rhp51Δ*	pHL2882	Wild-type Tf1-natAI	Sequencing of integration profile in rhp51Δ strain	This study
YHL12070	*h+ ade6-M216 ura4-D18 leu1-32 cwf12Δ*	pHL2882	Wild-type Tf1-natAI	Sequencing of integration profile in cwf12Δ strain	This study

Guo, Y., and H. L. Levin, 2010 High-throughput sequencing of retrotransposon integration provides a saturated profile of target activity in Schizosaccharomyces pombe. Genome Res 20**:** 239-248.

Kim, D. U., J. Hayles, D. Kim, V. Wood, H. O. Park *et al.*, 2010 Analysis of a genome-wide set of gene deletions in the fission yeast Schizosaccharomyces pombe. Nat Biotechnol 28**:** 617-623.

Levin, H. L., D. C. Weaver and J. D. Boeke, 1993 Novel gene expression mechanism in a fission yeast retroelement: Tf1 proteins are derived from a single primary translation product. Embo J 12**:** 4885-4895.

Lin, J. H., and H. L. Levin, 1997 A complex structure in the mRNA of Tf1 is recognized and cleaved to generate the primer of reverse transcription. Genes Dev 11**:** 270-285.

Sato, M., S. Dhut and T. Toda, 2005 New drug-resistant cassettes for gene disruption and epitope tagging in Schizosaccharomyces pombe. Yeast 22**:** 583-591.
